# High-fat diet impairs intermediate-term memory by autophagic-lysosomal dysfunction in *Drosophila*

**DOI:** 10.1371/journal.pgen.1011818

**Published:** 2025-08-18

**Authors:** Tong Yue, Minrui Jiang, Kotomi Onuki, Motoyuki Itoh, Ayako Tonoki

**Affiliations:** Department of Biochemistry, Graduate School of Pharmaceutical Sciences, Chiba University, Chiba, Japan; Universidad de Valparaiso, CHILE

## Abstract

High-fat diet (HFD) is considered a risk factor for age-related memory impairments such as Alzheimer’s disease. However, how HFD affects memory formation remains unclear. In this study, we established a model of memory defects caused by HFD in *Drosophila*. Our results revealed that the HFD impaired intermediate-term memory (ITM), but not short-term memory (STM), produced by classical aversive olfactory conditioning, and decreased autophagic activity in the heads of the HFD-fed flies. Transient reduction in autophagic activity also impaired ITM, but not STM. Genetic enhancement of autophagic activity in neurons effectively restored ITM performance in the HFD-fed flies. Mechanistically, HFD impairs lysosomal function by downregulating the expression of lysosome-related genes, leading to impaired fusion of autophagosomes with lysosomes. These findings suggest that HFD impairs ITM by reducing autophagic activity and lysosomal dysfunction in the neurons.

## Author summary

Recent research has shown that the high-fat diet (HFD) may increase the risk of memory problems and conditions like Alzheimer’s disease, but how these diets affect memory formation remains unclear. Our study used fruit flies as a simple model to explore how HFD influences memory. Our findings show that when fruit flies were fed HFD, it specifically impaired their intermediate-term memory (memory lasting several hours) while leaving short-term memory unaffected. We discovered that HFD reduces a crucial cellular cleaning process called autophagy in the brain, which normally helps remove cellular waste and maintain healthy function. When we enhanced this cleaning mechanism in the flies’ brains, the memory issues were reversed under HFD conditions. This study provides new insights into how diet influences memory and may contribute to future research on preventing or treating memory loss associated with aging and neurodegenerative diseases.

## Introduction

Dietary intake, such as a high-fat diet (HFD), is considered a risk factor for age-related memory impairments, such as Alzheimer’s disease (AD) [1–3]. HFD has been reported to cause learning and memory impairments in mouse models [4,5]. HFD-induced obesity exacerbates AD-related pathology and symptoms in mice [6]. However, the mechanisms of how HFD impairs memory remain unclear.

Recent studies have identified autophagy, a critical cellular recycling process, as a potential link between HFD and memory impairment [7,8]. Autophagy and lysosomal processes are critical in maintaining neuronal homeostasis by clearing damaged organelles and misfolded proteins [9–12]. Accumulating evidence suggests that impaired autophagy contributes to neurodegenerative diseases and cognitive deficits [13,14]. However, whether and how HFD impairs memory through reduced autophagic activity in neurons remains unclear. Additionally, it remains to be determined whether enhanced autophagic activity can mitigate HFD-induced memory impairment.

Current research predominantly relies on rodent models to investigate HFD cognitive effects [15,16] despite their significant limitations, such as high maintenance costs and genetic complexity [17,18]. Furthermore, most rodent studies have focused on specific regions, such as the hippocampus [16,19], leaving the broader impacts of HFD on the entire nervous system underexplored. In this study, we used *Drosophila* as a model system, which offers the following key advantages: conserved metabolic and neural pathways with mammals, a short lifespan, powerful genetic tools, and well-validated memory assessment protocols [20–22]. This model enables a more precise and comprehensive investigation of HFD-induced neurological alterations using targeted transgenic methods.

In this study, we examined the effects of HFD on memory performance in *Drosophila*, focusing on the role of autophagy and lysosomal function in the nervous system. We revealed that the HFD impairs intermediate-term memory (ITM) by disrupting autophagic activity through its impact on lysosomal function, whereas short-term memory (STM) remains unaffected. Notably, a transient reduction in autophagic activity within neurons was sufficient to impair ITM, whereas enhancing autophagic activity in neurons restored HFD-induced memory impairment. We also observed that the HFD disrupts lysosomal function with a reduced expression of lysosome-related factors, leading to impaired degradation of autophagic substrates. Overall, our study reveals that the autophagic–lysosomal system in neurons plays a vital role in diet-dependent memory changes.

## Results

### HFD impairs intermediate-term memory

**Fig 1:**
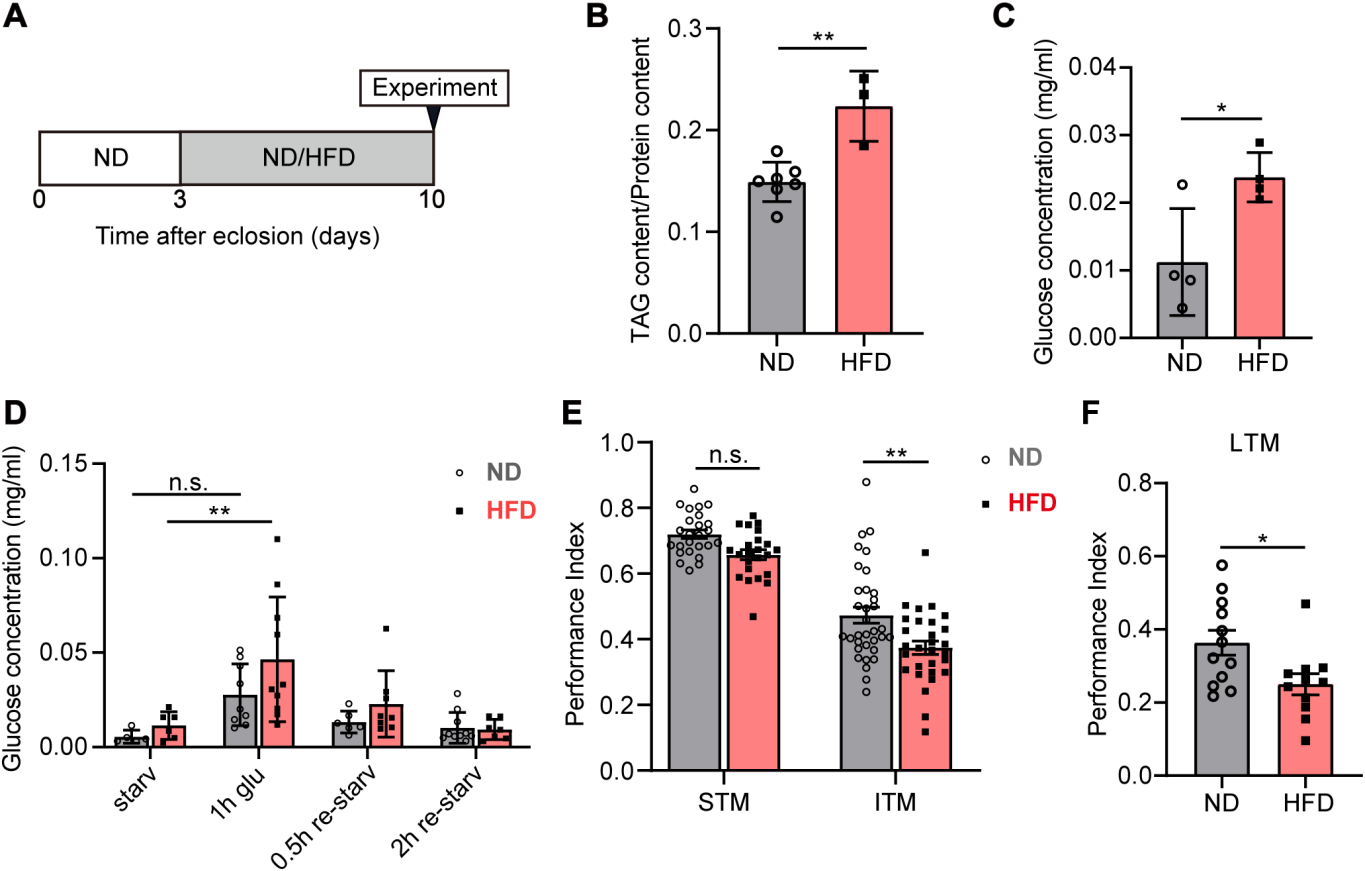
HFD decreases ITM and alters metabolic homeostasis. **(A)** Food treatment condition and feeding paradigm. The control diet (ND) and high-fat diet (HFD) were treated for 7 days in *Canton-S* flies. **(B)** Whole body triacylglycerol (TAG) levels in flies fed with ND and HFD. The TAG accumulated in the whole body significantly increased under the HFD condition compared to the ND condition. Error bars indicate SD (n = 7, 3. Student’s *t*-test, ** p < 0.01). **(C)** Relative circulating glucose levels. HFD-fed flies significantly increased basal glucose levels compared to ND-fed flies. Error bars indicate SD (n = 4. Student’s *t*-*t*est, * p < 0.05). **(D)** Hemolymph was collected from ND-fed and HFD-fed flies at each timepoint. HFD-fed flies showed a significant rise of blood glucose level immediately after glucose loading compared to the blood glucose level after starvation (n ≥ 5, Two-way ANOVA, ** p < 0.01), while ND-fed flies showed a moderate rise of glucose level. The error bars show standard deviation. **(E)** Short-term memory (STM) and intermediate-term memory (ITM) in 10-day-old flies fed with ND and HFD. ITM was significantly decreased in the HFD group. Error bars indicate SEM (n = 25, 23, 34, 29. Two-way ANOVA, n.s., not significant, ** p < 0.01). **(F)** Long-term memory (LTM) was significantly decreased in the HFD group. Error bars indicate SEM (n = 12, 11. Student’s t-test, * p < 0.05).

To examine the impact of HFD on energy metabolism, we collected three-day-old wild-type flies, maintained them under normal diet (ND) or HFD conditions for seven days, and then measured the triacylglycerol (TAG) and circulating glucose levels in their hemolymph (Fig 1A). The survival rate was not significantly affected after seven days of HFD feeding in wild-type male flies (S1A Fig). TAG and glucose levels significantly increased in HFD-fed flies (Fig 1B and 1C), which is consistent with previous studies [23,24]. Furthermore, HFD feeding significantly increased intestinal lipid accumulation, as determined by quantitative analysis of Oil Red O staining (S1B Fig). We also performed the glucose tolerance test to measure hemolymph glucose fluctuations by feeding flies a glucose solution after the starvation treatment [25]. In the glucose tolerance test, hemolymph was collected at four time points: after 24 h of starvation, after 1 h of 10% glucose feeding, and at 0.5 and 2 h following re-starvation after glucose exposure. After 1 h of glucose treatment, glucose levels increased in both ND-fed and HFD-fed flies; however, the elevation was significantly greater in the HFD-fed flies (Fig 1D). To determine whether the elevated glucose levels in HFD-fed flies following glucose loading were due to increased intake, we conducted a short-term feeding assay using blue dye during the 1-hour glucose feeding period. The results showed no significant difference in glucose intake between HFD-fed and ND-fed flies (S1C Fig), indicating that the elevated glucose levels in HFD-fed flies were not attributable to differences in food consumption. These data suggest that HFD alters metabolic homeostasis.

To examine dietary effects on memory in *Drosophila*, we tested their memory performance using classical olfactory conditioning. STM, tested 3 min after conditioning, was not affected by the HFD (Fig 1E, left). ITM, tested 3 h after conditioning, was markedly reduced in the HFD-fed flies (Fig 1E, right). The HFD did not modify the avoidance of electric shocks in flies (S1D Fig). Odor perception in 3-octanol (Oct) did not change, whereas odor perception in benzaldehyde (Benz) significantly decreased in the HFD-fed flies (S1E Fig). As STM did not change despite the decreased perception of Benz, and ITM decreased in the HFD-fed flies, these results suggest that the HFD impaired ITM. To exclude the potential effects of altered odor perception on memory performance in the HFD-fed flies, we used 4-methylcyclohexanol (MCH) as an alternative odor to Benz to assess the effect of HFD on memory performance. Odor avoidance tests revealed that HFD-fed and ND-fed flies exhibited comparable avoidance responses to MCH (S1F Fig). We then assessed memory performance using MCH and Oct as olfactory cues. Consistent with the Benz results, HFD feeding did not affect STM but significantly impaired ITM in flies (S1G Fig). These results supported that HFD-induced ITM deficits are not attributable to changes in odor sensitivity.

To further investigate the impact of HFD on long-term memory (LTM) performance, we assayed memory at 24 h after 5x spaced training [26]. The results demonstrated that HFD-fed flies exhibited significantly impaired LTM compared to ND (Fig 1F). These results indicated that HFD feeding negatively impacts multiple phases of memory formation.

### HFD feeding impairs autophagic activity in brains

**Fig 2:**
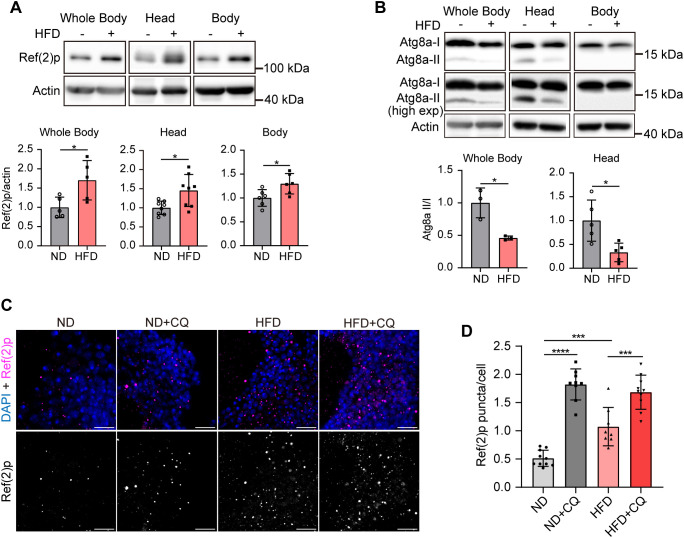
HFD impairs autophagic activity in fly brains. **(A)** HFD significantly increased the protein levels of Ref(2)p protein in the whole body, head, and body samples. The quantification of band intensity is shown below. Error bars indicate SD (n ≥ 5. Students’ *t*-test, * p < 0.05). **(B)** Western blot analysis of Atg8a lipidation in the samples of whole body, head, and body of ND or HFD-fed flies. The lower band represents lipidated Atg8a (Atg8a-II), and the upper band represents non-lipidated Atg8a (Atg8a-I). To obtain clearer Atg8a-II protein bands, the images were subjected to high-exposure processing (high exp). The high-exposure processed bands were placed below the original figures. The quantification of band intensity is shown below. Error bars indicate SD (n ≥ 3. Students’ *t*-*t*est, * p < 0.05). **(C)** Representative images of Ref(2)p immunostaining in the area of cell bodies of mushroom body neurons (MBNs) at 10-day-old *Canton-S* flies fed with ND, ND + CQ, HFD, and HFD + CQ. Scale bar: 10µm. **(D)** Quantification of Ref(2)p puncta. Error bars indicate SD (n = 10, 9, 8, 10. One-way ANOVA, *** p < 0.001, **** p < 0.0001).

An increasing number of studies have shown that HFD impairs hepatic autophagy [27,28]. Moreover, various studies have demonstrated that HFD leads to cardiac damage by suppressing autophagy [29,30]. Recent evidence has suggested an association between autophagy and cognitive performance [31,32]. However, it remains unclear whether HFD impairs memory via autophagic dysfunction. Therefore, we investigated the impact of HFD on autophagy in the whole body, head, and body (thorax and abdomen) of flies by quantifying autophagy-related protein levels using Western blot analysis. Ref(2)p serves as an autophagy receptor that is degraded during autophagy [33]. Therefore, Ref(2)p accumulation reflects the autophagic dysfunction. The protein level of the autophagy substrate Ref(2)p significantly increased in the whole body, head, and body samples of HFD-fed flies (Fig 2A). In *Drosophila*, *atg8a* is the ortholog of mammalian LC3 and yeast *atg8a*. Atg8a (Atg8a-I) covalently binds to phosphatidylethanolamine during autophagosome formation and is transformed into membrane-type lipidated Atg8a (Atg8a-II), which localizes to the autophagic membrane [34–36]. Atg8a is involved in autophagosome formation and is crucial for substrate recognition during selective autophagy [37]. The level of autophagic activity can be estimated by the magnitude of the Atg8a-II/I ratio. HFD-fed flies showed a lower ratio of Atg8a-II/I than ND-fed flies in the whole body and head samples (Fig 2B). Protein bands of Atg8a-II were not observed in the body lysates (Fig 2B). These results suggest that HFD-fed flies exhibit autophagic dysfunction primarily in the head, rather than in the body.

To investigate whether HFD feeding impairs autophagic degradation in the brain, we analyzed Ref(2)p accumulation by immunohistochemistry in the area of mushroom body neurons (MBNs). Kenyon cells (KCs) in the MBNs of fruit flies are the primary neurons involved in learning and memory [38–40]. Flies fed the HFD for seven days showed an increased number of Ref(2)p compared to ND-fed flies (Fig 2C and 2D). As a control for autophagy inhibition, flies were treated with 2.5 mg/mL chloroquine (CQ) for 24 h before dissection. CQ is a classical autophagy inhibitor that accumulates in lysosomes, neutralizing their acidic pH and inhibiting autophagosome–lysosome fusion and proteolytic degradation [41,42]. As expected, CQ treatment led to significant accumulation of Ref(2)p in ND flies (ND + CQ) (Fig 2C and 2D). Notably, CQ treatment further elevated Ref(2)P levels in HFD-fed flies (HFD + CQ), although the increase was less pronounced than that observed in ND controls (Fig 2C and 2D). These results indicate that HFD mildly impairs autophagic function, although the specific step in the pathway remains to be clarified.

### Transient suppression of autophagy in neurons impairs ITM

**Fig 3:**
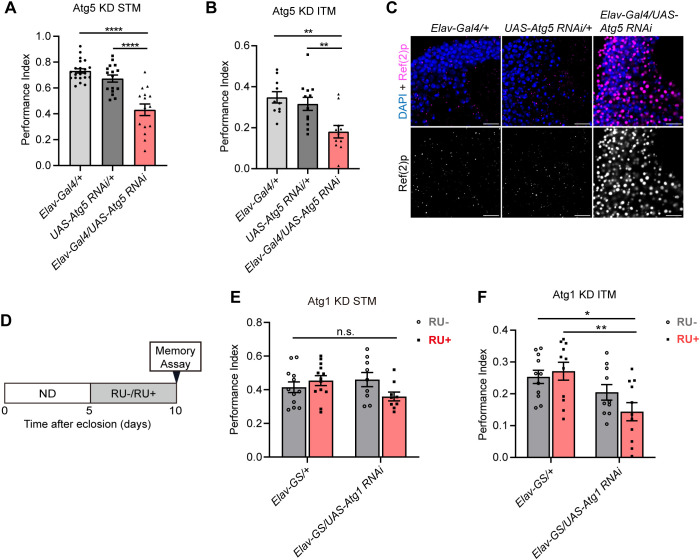
Transient suppression of autophagy in neurons impairs ITM. **(A)** STM was significantly impaired in *Elav-Gal4/UAS-Atg5 RNAi* flies compared to control groups. Error bars indicate SEM (n = 22, 16, 15. One-way ANOVA, **** p < 0.0001). **(B)** ITM was significantly impaired in *Elav-Gal4/UAS-Atg5 RNAi* flies compared to control groups. Error bars indicate SEM (n = 10, 12, 10. One-way ANOVA, ** p < 0.01). **(C)** Representative images of Ref(2)p immunostaining in the cell body area of MBNs of *Elav-Gal4/UAS-Atg5 RNAi* and control groups. Confocal sections through the MBNs were shown. Scale bar: 10µm. **(D)** The experimental timeline shows the time of RU486 feedings and memory tests in 10-day-old flies. **(E)** STM in flies with transient knockdown of Atg1 in neurons (*Elav-GS/UAS-Atg1-RNAi* RU+) was not changed compared with other genotypes. Error bars indicate SEM (n = 12, 13, 9, 9. Two-way ANOVA, n.s., not significant). **(F)** The ITM was significantly reduced in flies with transient knockdown of Atg1 (RU+) flies compared to control groups. Error bars indicate SEM (n = 11, 11, 10, 11. Two-way ANOVA, * p < 0.05, ** p < 0.01).

To test whether autophagic dysfunction affects memory formation, memory was examined when Atg5, which is a key protein involved in autophagosome membrane extension [43,44], was knocked down by RNA interference (RNAi) in fly neurons (*Elav-Gal4/UAS-Atg5 RNAi*). The Atg5 RNAi expression in whole body showed approximately 90% knockdown efficiency (*Tub-Gal4/UAS-Atg5 RNAi*) (S2A Fig), and approximately 60% reduction in *atg5* expression was observed in fly heads with Atg5 RNAi expression in neurons (*Elav-Gal4/UAS-Atg5 RNAi*) (S2B Fig). As in previous studies [45], pan-neuronal inhibition of Atg5 significantly decreased STM and ITM in 10-day-old flies (Fig 3A and 3B). Ref(2)p accumulation was significantly elevated in the heads of Atg5 knockdown flies (Fig 3C). The knockdown of Atg5 in neurons did not affect odor perception but slightly decreased the electric shock response (S2C–S2E Fig), which may cause defects in STM performance.

Subsequently, to examine whether autophagy regulates memory in neurons without affecting development, we specifically attenuated autophagy in fly neurons by expressing an RNAi construct targeting Atg5 via a spatiotemporally inducible gene-switch (GS) system (*Elav-GS/UAS-Atg5 RNAi*) (S2F Fig) [46]. The gene is supposed to be knocked down in neurons only after flies were fed RU486 (RU) in the GS system (*Elav-GS)*. As previously reported, the GS system may exhibit a degree of leaky expression even in the absence of RU [47]. We observed such leaky expression of **Atg5* RNAi*, which led to a significantly reduced expression of *atg5* even without RU administration (S2F Fig). The results of memory assay showed that neuronal knockdown of *atg5* using the GS system did not affect ITM (S2G Fig). We next used RNA interference to transiently knock down *atg1* in the nervous system to suppress autophagic activity (S3A Fig). Atg1 serves as the most upstream regulator in the autophagy pathway and mediates autophagy initiation [48,49]. When *atg1* KD flies were fed RU-containing food for five days, their STM did not differ significantly from that of the control groups, whereas they exhibited impaired ITM (Fig 3D–3F). Transient *atg1* knockdown did not affect odor perception or electric shock avoidance in flies (S3B–S3D Fig). Our results indicate that transient inhibition of autophagy in neurons specifically impairs ITM, but not STM.

To investigate whether inhibition of autophagy specifically in neurons required for ITM leads to a reduction in ITM, we focused on Dorsal Paired Medial (DPM) neurons. DPM neurons innervate the mushroom bodies and play a critical role in the regulation of ITM [50,51]. To examine whether autophagic activity in DPM neurons is required for ITM maintenance, we selectively knocked down *atg1* in DPM neurons using the *VT64246-Gal4* driver, which is a specific driver for DPM neurons with no detectable expression elsewhere in the brain [52]. Knockdown of *atg1* in DPM neurons significantly impaired ITM (S3E Fig). The knockdown of Atg1 in DPM neurons did not affect the electric shock response and odor perception to benzaldehyde (Benz) but exhibited a slight reduction in odor perception to octanol (Oct) (S3F–S3H Fig). These results suggested that suppression of autophagic activity in DPM neurons impairs ITM.

### Elevated autophagic levels in neurons restore HFD-induced memory impairment

**Fig 4:**
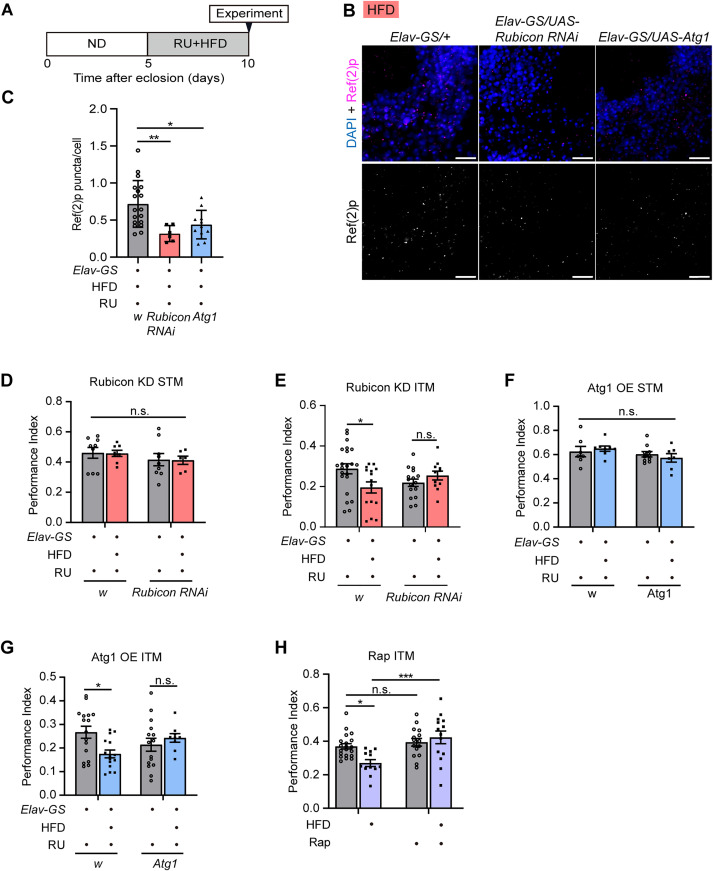
Elevated autophagic levels in neurons restore HFD-induced memory impairment. **(A)** The experimental timeline shows the time of RU + HFD feedings and memory tests in 10-day-old flies. **(B)** Representative images of Ref(2)p immunostaining in brains of 10-day-old *Elav-GS/ + *, *Elav-GS/UAS-Rubicon RNAi*, and *Elav-GS/UAS-Atg1* fed with RU + HFD. Confocal sections of the area of MB cell bodies were shown. Scale bar: 10µm. **(C)** Quantification of Ref(2)p puncta. Error bars indicate SD (n = 18, 6, 11. One-way ANOVA, * p < 0.05, ** p < 0.01). **(D)** The STM performance of *Elav-GS/+* and *Elav-GS/UAS-Rubicon-RNAi* flies. Error bars indicate SEM (n = 9, 8, 9, 6. Two-way ANOVA, n.s., not significant). **(E)** The ITM performance of *Elav-GS/+* and *Elav-GS/UAS-Rubicon-RNAi* flies. HFD induces memory decline in control groups but not in Rubicon knockdown flies. Error bars indicate SEM (n = 22, 15, 16, 11. Two-way ANOVA, * p < 0.05, n.s., not significant). **(F)** The STM performance of *Elav-GS/+* and *Elav-GS/UAS-Atg1* flies. Error bars indicate SEM (n = 7, 8, 10, 7. Two-way ANOVA, n.s., not significant). **(G)** The ITM performance of *Elav-GS/+* and *Elav-GS/UAS-Atg1* flies. HFD induces memory decline in control groups but not in Atg1 overexpression flies. Error bars indicate SEM (n = 16, 15, 15, 9. Two-way ANOVA, * p < 0.05, n.s., not significant). **(H)** The ITM performance in 10-day-old flies fed with ND, ND+Rapamycin (Rap), HFD, and HFD + Rap. Error bars indicate SEM (n = 20, 15, 12, 14. Two-way ANOVA, * p < 0.05, ***p < 0.001, n.s., not significant).

Subsequently, we addressed whether elevated levels of autophagy in neurons could ameliorate HFD-induced memory decline. We enhanced autophagic activity in neurons while feeding them with HFD and then measured their ITM. Rubicon, a negative regulator of autophagy, inhibits autophagosome-lysosome fusion process [53,54]. Rubicon knockdown enhances autophagosome maturation and endocytosis [55]. First, we examined whether HFD induces an upregulation of *rubicon* expression. The expression of *rubicon* was significantly increased in the heads of HFD-fed flies (S4A Fig). Five-day-old *rubicon* knockdown flies were maintained on RU-containing ND or HFD for five days, after which their memory performance was assessed (Fig 4A). qPCR showed that the silencing efficiency of *rubicon* knockdown was approximately 60% in flies expressing *rubicon* RNAi in neurons using the GS system (S4B Fig). A leaky expression of *rubicon* was also observed in the absence of RU. We also examined the changes in autophagy in the brain after neuronal knockdown of *rubicon*. In *rubicon* knockdown flies, the amount of Ref(2)p was significantly reduced compared to that in the control group under either ND or HFD conditions (Figs 4B and 4C and S5), suggesting that *rubicon* knockdown increased autophagic activity in the brain. The results of the memory experiment showed that STM did not change when *rubicon* was knocked down in the neurons (Fig 4D). Although HFD caused ITM decline in the control group, HFD-induced ITM decline was suppressed in *rubicon* knockdown fruit flies (Fig 4E). Furthermore, odor and shock acuity did not change in *rubicon* knockdown flies (S6A–S6C Fig). These results indicate that the *rubicon* knockdown followed by an increase in autophagic activity in neurons, restores HFD-induced memory deficits.

We also used flies with *atg1* overexpression to enhance autophagic activity in neurons. Previous studies have shown that overexpression of *atg1* is sufficient to induce high levels of autophagy [56]. qPCR results showed an approximately five-fold increase in *atg1* mRNA levels in the heads of *Elav-GS/UAS-Atg1* flies under RU-mediated transgenic induction (S4C Fig). Atg1 overexpression significantly decreased the amount of Ref(2)p compared to the control group under either ND or HFD conditions (Figs 4B and 4C and S5B and S5C). These results suggest that Atg1 overexpression in neurons increases autophagy in the brain. We subsequently measured memory in Atg1 overexpressing flies under HFD feeding. There was no significant difference in the STM when *atg1* was overexpressed in neurons (Fig 4F). However, the HFD-induced ITM decline was suppressed by *atg1* overexpression (Fig 4G). Odor and shock acuity remained unaffected in these flies (S6D–S6F Fig).

To confirm these results, the autophagy inducer rapamycin (Rap) was used to enhance autophagic activity in flies. The kinase mammalian target of rapamycin (mTOR) negatively regulates autophagy processes [57]. Rap is an mTOR inhibitor that activates autophagy by inhibiting mTORC1 [58,59]. Rap was fed to flies along with HFD to test whether it affected memory. Although the addition of Rap to the ND did not affect ITM, the addition of Rap to the HFD significantly restored ITM compared with that in the HFD-only group (Fig 4H). These findings indicate that memory deficits induced by HFD are reversible and that enhanced autophagic activity effectively alleviates HFD-induced ITM impairment.

### HFD changes lysosomal activity in the autophagy process

**Fig 5:**
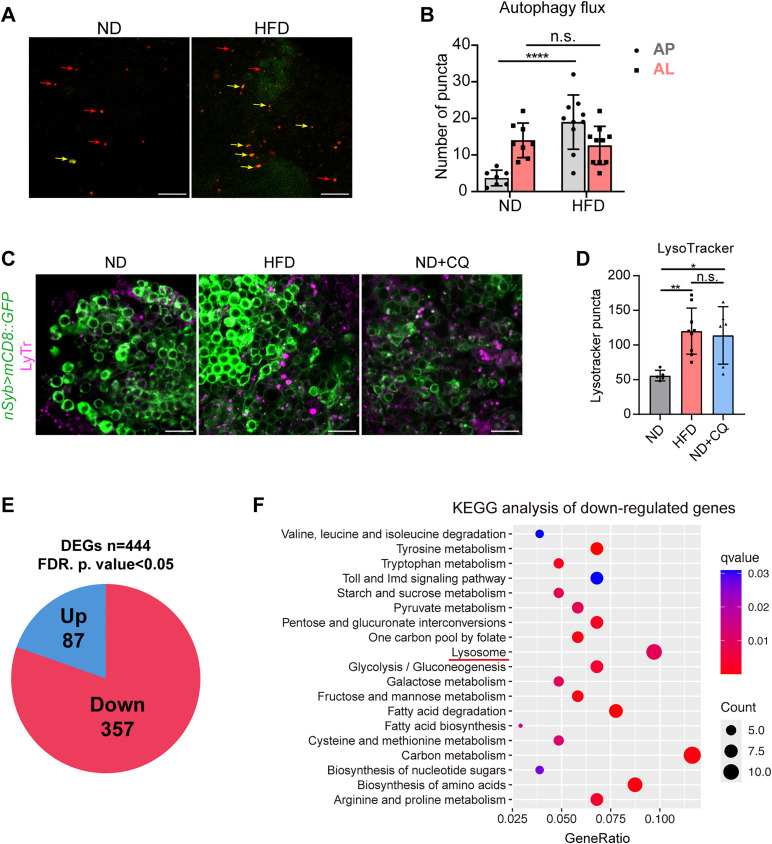
HFD changes lysosomal activity. **(A)** Representative images of Kenyon cell layers of flies expressing the UAS-GFP-mCherry-Atg8a transgene exclusively in the neuron systems (Elav-GS). The autophagy flux in Kenyon cell layers of brains suggested that HFD increased autophagosomes (AP, yellow arrows), whereas the number of autophagic lysosomes (AL, red arrows) remained unchanged. **(B)** Quantification of mCherry-GFP-Atg8a puncta. Error bars indicate SD (n = 10, 9, 8, 10. Two-way ANOVA, n.s., not significant, **** p < 0.0001). **(C)** Representative images of LysoTracker Red staining in MB at 10-day-old flies fed with ND, HFD, and ND + CQ. Lysosomes were marked by the LysoTracker Red signal. Confocal sections through the cell body of MBNs were shown. Scale bar: 10µm. **(D)** Quantification of LysoTracker Red in Kenyon cell layers. Error bars indicate SD (n = 5, 9, 6. One-way ANOVA, *p < 0.05, **p < 0.01, n.s., not significant). **(E)** Pie chart illustrating the distribution of 444 differentially expressed genes (DEGs), including 87 upregulated genes and 357 downregulated genes. **(F)** KEGG analysis of downregulated genes in ND and HFD head samples.

We next sought to determine how HFD affects autophagic activity at the cellular level. To quantify autophagic flux, we employed the dual-fluorescent mCherry-GFP-Atg8a reporter, which is widely used to monitor autophagy dynamics [60]. In this system, autophagosomes are visualized as yellow puncta (dual GFP + /mCherry+ labeling), whereas autolysosomes appear as red puncta (mCherry + /GFP–) because the acidic pH of lysosomes selectively quenches GFP fluorescence while preserving mCherry signal. HFD-fed flies exhibited an increased number of autophagosomes without a corresponding increase in autolysosomes, indicating impaired autophagic flux (Fig 5A and 5B).

To further elucidate the specific mechanism underlying HFD-induced impairment of autophagic flux, we next examined lysosome abundance. Lysosomes play a central role in autophagy [61–63]. In the final stage of autophagy, the autophagosome fuses with the lysosome to form the autolysosome, where all the contents are eventually degraded [62,64]. Given the significant increase in autophagosome numbers without a corresponding change in autolysosomes, we next investigated whether this reflected insufficient lysosomal capacity or a defect in autophagosome maturation. We stained the dissected brains with the fluorescent dye LysoTracker Red DND-99 (LyTr) to observe the number of lysosomes in Kenyon cells of the MB, which preferentially accumulate in vesicles with an acidic pH. CQ was used as a positive control, as it is known to induce lysosomal accumulation. As expected, CQ treatment significantly increased the LyTr-positive signal (Fig 5C and 5D). Interestingly, HFD feeding also resulted in a marked increase in lysosomal abundance in Kenyon cells, comparable to that observed in the CQ group (Fig 5C and 5D). The concurrent accumulation of autophagosomes and lysosomes, without a corresponding increase in autolysosomes, suggests that the impaired autophagic flux under HFD conditions is unlikely due to insufficient lysosomal capacity. Instead, these findings point to a potential defect in the fusion process between autophagosomes and lysosomes, preventing the formation of functional autolysosomes despite the availability of both compartments. Together, these results indicate that the impaired autophagic flux observed under HFD conditions is attributable to a defect in autophagosome–lysosome fusion.

To gain further insights into the molecular mechanisms underlying this defect, we performed RNA sequencing on heads from ND and HFD-fed flies. Differential gene expression analysis identified a total of 444 genes with significant changes in expression (FDR-adjusted p-value < 0.05), including 87 upregulated and 357 downregulated genes in the HFD-fed flies (Fig 5E) (S1 Data), based on the threshold of log2(fold change) ≥ 0.5 or ≤ −0.5. Kyoto Encyclopedia of Genes and Genomes (KEGG) pathway analysis of the downregulated gene set revealed significant enrichment of pathways such as lysosome and carbon metabolism, suggesting that lysosomal signaling is markedly downregulated in HFD-fed flies (Fig 5F). These results indicated that HFD may disrupt autophagic flux through compromised lysosomal function, potentially contributing to the observed memory impairments.

### Inhibition of lysosomal activity leads to memory impairment

**Fig 6:**
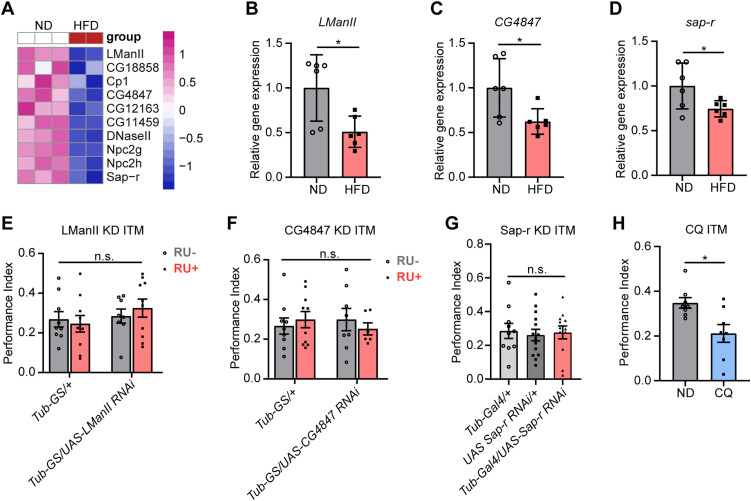
Inhibition of lysosomal activity causes memory impairment. **(A)**Heatmap of significantly down-regulated genes associated with the lysosomal signaling pathway between ND and HFD. **(B–D)** HFD-fed fly heads showed decreased expression of *LManII* **(B)**, *CG4847* **(C)**, and *sap-r* **(D)**. qPCR measures each gene’s relative expression levels in ND and HFD head samples. The normalized expression level is shown (n = 6. Mann-Whitney test for *LManII*; other genes use Student’s *t*-test, * p < 0.05). **(E)** The ITM performance in flies with LManII knockdown in the whole body (*Tub-GS/UAS-LManII RNAi*, RU+) is unchanged compared with other groups. Error bars indicate SEM (n = 9, 10, 8, 10. Two-way ANOVA, n.s., not significant). **(F)** The ITM performance in flies with CG4847 knockdown in the whole body (*Tub-GS/UAS-CG4847 RNAi*, RU+) is unchanged compared with other groups. Error bars indicate SEM (n = 9, 10, 8, 6. Two-way ANOVA, n.s., not significant). **(G)** The ITM performance in flies with sap-r knockdown in the whole body (*Tub-Gal4/UAS Sap-r RNAi*) is unchanged compared to other groups. Error bars indicate SEM (n = 8, 14, 12. Two-way ANOVA, n.s., not significant). **(H)** The ITM performance was significantly reduced in CQ-fed flies compared to control groups. Error bars indicate SEM (n = 8. Student’s *t*-*t*est, * p < 0.05).

To further explore the contribution of lysosomal dysfunction, we examined whether individual lysosome-related genes would affect memory performance. According to the KEGG database analysis, 10 of the 357 significantly down-regulated genes were associated with the lysosomal signaling pathway (Fig 6A). Among the 10 genes, qPCR validation of three genes, *LManII*, *CG4847*, and *sap-r*, confirmed the RNA sequencing results. These genes exhibited a significant reduction in expression in the HFD group (Figs 6B–6D and S7). We subsequently assessed memory performance following knockdown of these three genes (S8A–S8D Fig). As conditional knockdown of sap-r using the *Tub-GS* driver did not achieve sufficient gene suppression (S8C Fig), we employed the *Tub-Gal4* driver in subsequent memory experiments to ensure more effective knockdown (S8D Fig). The knockdown of a lysosome-related gene alone did not result in significant memory impairments (Fig 6E–6G). Finally, we evaluated ITM after five days of rearing with the lysosomal inhibitor CQ to examine whether broader lysosomal dysfunction could impact memory. The CQ treatment did not affect the odor or shock acuity of the flies (S8E and S8F Fig). The ITM of CQ-fed flies was significantly decreased compared to that of the ND-fed flies (Fig 6H). These findings suggest that inhibition of lysosomal activity contributes to ITM impairment.

## Discussion

Our findings indicate that HFD impairs memory performance in *Drosophila*, which is associated with a significant decrease in neuronal autophagic activity. Enhancing autophagic activity in neurons alleviates HFD-induced memory impairment. Furthermore, our experiments revealed that lysosomal function was compromised in the HFD-fed flies. Overall, these findings highlight the critical roles of autophagy and lysosomal function in the maintenance of memory performance under HFD-induced metabolic stress (S9 Fig).

In recent years, growing evidence has shown that HFD impairs cognitive function by inhibiting autophagic degradation [65,66]. Consequently, it is critical to explore the mechanisms underlying this impairment and to determine whether enhanced autophagic activity can mitigate HFD-induced memory decline. Our research found that HFD reduced Atg8a conversion and led to a significant accumulation of Ref(2)p in brains, causing ITM decline. Numerous studies have shown that transmission from MB neurons is essential for olfactory memory [67–69]. Previous studies have shown that autophagy regulates synaptic formation and function. Autophagy-deficient flies exhibit reduced size and number of synaptic boutons and decreased neurotransmitter release [70,71]. HFD-induced decrease in autophagic flux may impair synaptic function, leading to memory decline. Our study concluded that HFD-induced memory impairment was reversible, as it was ameliorated by enhancing autophagic activity in neurons.

We also found that Atg5 knockdown in neurons decreased both the STM and ITM, as previously reported [8,45]. This may be attributed to the inhibition of neuronal autophagic activity during development. Loss of Atg5 function leads to imbalanced differentiation and proliferation of cortical neural progenitor cells, resulting in abnormal cortical neuronal morphology [72]. Disruption of autophagy during development may affect neural development, leading to significantly worse brain function, including conformational memory, compared to the control group. In contrast, temporary pan-neuronal inhibition of *atg1* in neurons specifically impaired the decline in ITM without significantly affecting STM. This suggests that autophagy is indispensable for ITM maintenance, even in the fully developed nervous system and highlights its distinct roles in different types of memory formation.

The differential effects on ITM observed between transient Atg1 (Fig 3F) and Atg5 (S2F Fig) knockdown using the *Elav-GS* system may reflect their distinct roles in the autophagic process. Atg1 functions as a key initiator of autophagy [48,49]. Therefore, its inhibition may lead to a rapid and substantial reduction in autophagosome formation, resulting in acute impairment of autophagic activity. In contrast, Atg5 is primarily involved in autophagosome membrane elongation and closure [73]. Transient knockdown of Atg5 may only partially disrupt autophagosome maturation, and residual Atg5 protein may be sufficient to maintain basal autophagic activity for a short period. Consequently, Atg5 knockdown using the *Elav-GS* system may not significantly affect memory performance.

While Rubicon knockdown or Atg1 overexpression effectively alleviated HFD-induced memory decline, both manipulations led to a modest reduction in memory performance under normal dietary conditions. These findings suggest that although enhancing autophagy can be beneficial in conditions where autophagic activity is suppressed (such as under HFD), excessive activation of autophagy in a physiologically normal context may be detrimental to neuronal function and memory [74,75]. The slight impairment in intermediate-term memory (ITM) observed in Rubicon RNAi or Atg1-overexpressing flies on a normal diet may therefore reflect autophagy-associated cytotoxicity or disruption of homeostatic neuronal processes. In contrast, under HFD conditions, where autophagic flux is impaired, these genetic interventions may restore autophagy to an optimal level, thereby rescuing memory performance. Compared to these direct genetic manipulations, rapamycin-induced activation of autophagy may be more moderate, which could explain the absence of adverse memory effects under normal dietary conditions.

Moreover, we observed that the HFD similarly impaired ITM in *Drosophila* without affecting STM. Interestingly, HFD-fed flies exhibited reduced avoidance of Benz, consistent with previous reports that HFD alters olfactory sensitivity by downregulating Or43a, a gene encoding one of the benzaldehyde receptors [76,77]. However, this olfactory decline cannot account for ITM impairment, as the STM in flies remained unaffected. STM in *Drosophila* forms rapidly after training and relies primarily on transient synaptic plasticity, which involves modifications of pre-existing proteins and altered neurotransmitter release without requiring new protein synthesis [78,79]. In contrast, intermediate-term memory (ITM) persists for several hours and requires more sustained neural remodeling and protein turnover [50,80]. Given the essential role of autophagy in protein quality control, organelle clearance, and the maintenance of synaptic homeostasis [81], ITM may be more vulnerable to impaired autophagic flux. Therefore, we propose that HFD-induced ITM deficits may be linked to inhibition of autophagic activity in DPM and MB neurons, which are critical for ITM regulation. Supporting this hypothesis, we found that selective Atg1 knockdown in DPM neurons using the *VT64246*-*Gal4* driver significantly impaired ITM, indicating that autophagic activity in DPM neurons is required for ITM maintenance. It is plausible that HFD disrupts ITM integrity by suppressing autophagic activity in specific neuronal populations, such as DPM neurons. Elucidating the exact mechanisms by which HFD interferes with neuronal autophagy is crucial to understanding its impact on memory regulation.

Lysosomes, which contain more than 60 acidic hydrolases, serve as the primary degradation compartments in eukaryotic cells [82]. Autophagy depends on the degradative function of lysosomes to facilitate breakdown and recycling of cellular components, thereby maintaining cellular homeostasis [83]. A growing body of evidence has indicated that lysosomal dysfunction is associated with various neurodegenerative diseases [12,84]. In this study, we found that the brains of the HFD-fed flies exhibited increased LyTr intensity. RNA sequencing further revealed a significant enrichment of lysosomal signaling pathways among genes with reduced expression in the HFD-fed flies, which aligns with previous findings that lysosomal dysfunction plays a pivotal role in neurodegenerative conditions and autophagic impairment [85–87]. Lysosomes are essential for autophagic flux, as they facilitate the degradation of autophagosomal cargo through fusion with autophagosomes [62]; therefore, disruptions in lysosomal function can directly hinder this process. Consistent with these results, our results suggest that HFD impairs lysosomal function with decreased expression of some lysosomal factors in the brain, leading to defective autophagic flux, likely due to impaired autophagosome-lysosome fusion. Additionally, direct feeding of the lysosomal inhibitor CQ resulted in memory deficits. While knockdown of individual lysosomal genes (*LManII*, *CG4847*, and *sap-r*) did not affect ITM performance (Fig 6E–6G), pharmacological inhibition of lysosomal function by CQ significantly impaired memory (Fig 6H). This difference likely reflects the extent of lysosomal impairment: single gene knockdowns may only partially disrupt specific lysosomal functions, allowing compensatory mechanisms to preserve overall lysosomal activity. In contrast, CQ broadly impairs lysosomal degradation by increasing lysosomal pH and inhibiting autophagosome-lysosome fusion [41,42], thereby exerting a stronger effect on autophagic flux and memory. These findings demonstrate the critical role of lysosomal dysfunction in impairing autophagic flux and contributing to memory deficit. Previous studies have demonstrated that enhancing lysosomal function can rescue HFD-induced pathophysiological deficits [88,89]. However, the exact mechanism underlying HFD-induced lysosomal dysfunction requires further investigation.

## Materials and methods

### Fly stocks and husbandry

Flies were maintained at 25°C, 70% relative humidity with a constant 12 h:12 h light-dark cycle. Flies were fed on standard *Drosophila* food unless otherwise specified. Approximately 250 flies born in 2–3 days were raised in food bottles and transferred to fresh food every 3 or 4 days until the desired age was reached. *Canton-S* was used as the wild-type strain. *Tubulin-GeneSwitch (GS)* flies were kindly provided by Dr. Scott Pletcher (Michigan Neuroscience Institute). *Elav-GS* (#*43642*)*, Elav-Gal4 (#25750), nSyb-Gal4 (#51941), Tubulin-Gal4 (#5138), UAS-Atg1 RNAi (#44034), UAS-Atg5 RNAi (#34899), UAS-Rubicon RNAi (#43276), UAS-Atg1 (#51655), UAS-mCD8::GFP (#51944),* and *UAS-GFP-mCherry-atg8a (#37749)* were obtained from the Bloomington *Drosophila* Stock Center. The following stocks were obtained from the Vienna *Drosophila* Resource Center: *UAS-LManII RNAi (#GD49352), UAS-CG4847 RNAi (#GD3231), UAS-Sap-r RNAi (#GD51130),* and *VT64246-Gal4 (v204311)* [52]*.*

### Fly food

For the high-fat-feeding experiments, flies at 3 days of age after eclosion were collected and fed with standard fly food (normal diet, ND) (8% glucose) or high-fat diet (HFD) (8% glucose + 20% coconut oil (25605–75, Nacalai)) for 7 consecutive days. For the GS experiment, RU486 (RU) was administered at a final concentration of 200 μM. We added 20% coconut oil to RU food to make HFD supplemented with RU. Flies were fed RU-containing food for 5 days.

For rapamycin (Rap) (R-5000, LC Laboratories) treatment, a 50 mg/ml Rap stock in ethanol was added to standard fly food to reach a final concentration of 200 μM. Flies were fed for 7 days before experiments.

In immunohistochemistry experiments, flies were kept with 2.5 mg/mL of chloroquine (CQ) for 24 h. To examine the ITM in the CQ-fed flies, 5-day-old flies were exposed to food containing 1 mg/ml of CQ for 5 days, followed by conducting memory experiments.

### Learning and memory assay

The learning and memory assay was conducted under dim red light at 23°C and 70% relative humidity. Olfactory cues consisted of either 0.12% 3-Octanol (Oct) (O0121, TCI) paired with 0.08% Benzaldehyde (Benz) (B1334-2G, Sigma), or 0.15% 3-Octanol paired with 0.10% 4-methylcyclohexanol (MCH) (589-91-3, TCI). A 90V alternating current was used as the behavioral reinforcer. Fifty to sixty flies were placed in an electronic shock tube and sequentially exposed to one odor (CS+) paired with an electric shock for 1 min, followed by a 30-sec rest, and then to a second odor (CS-) without an electric shock for 1 min. To assess STM, the flies were immediately transferred to a T-maze, where they were allowed 2 min to choose between an arm with the CS+ odor and an arm with the CS- odor. For ITM, the flies were tested 3 h after training. For long-term memory (LTM) assay, flies were subjected to five training cycles with 15-minute intervals. After training, flies were returned to feeder vials and maintained at 23 °C. Memory performance was assessed 24 hours later.

The performance index (PI) for each odor was calculated as follows.


PI = (X−Y) / (X+Y)

X: the number of flies that chose the CS- odor

Y: the number of flies that chose the CS+ odor

The overall PI was then calculated as the average of the two reciprocal performance indices obtained by switching the odor-shock pairings.

In all experiments for learning and memory assay, n refers to the number of biological replicates, with each replicate consisting of 50–60 flies included per replicate. The PI for each replicate was calculated based on the average response of all flies within that group.

### Olfactory acuity

Approximately fifty to sixty flies were placed in a T-maze and given 2 min to choose between an odor (Oct, Benz, or MCH) in mineral oil on one side and fresh air passed through mineral oil without odor on the other side. The avoidance index (AI) was calculated as follows.


AI = (X−Y)/ (X+Y)

X: the number of flies that chose the mineral oil

Y: the number of flies that chose the odor

### Electric shock acuity

Approximately fifty to sixty flies were placed in a T-maze and given 2 min to choose between an arm with 12 pulses of electric shock at 90V and an arm without electric shock. The avoidance index (AI) was calculated as follows.


AI = (X−Y) / (X+Y)

X: the number of flies that chose no electric shock

Y: the number of flies that chose electric shock

### Hemolymph collection and glucose concentration measurement

The hemolymph glucose concentration was measured using the previously described method [90]. Female flies were used for hemolymph collection due to their larger volume, which facilitate more efficient and reliable sampling. The heads of 30–40 female *Canton-S* flies were severed, and hemolymph was collected by gently pressing the thorax using a 0.5 μl capillary (DRM 1-000-0005, Drummond). The hemolymph samples were then transferred to 96-well plates (0.25 μl per well). A glucose assay reagent (0.25 μl per well) (GAHK20–1KT, Sigma) was added to each well. For the glucose standard curve, 5 μl of glucose standard (GAHK20–1KT, Sigma) and a blank (PBS) were added to each well, followed by 45 μl of glucose assay reagent. The absorbance was measured at 340 nm using a FilterMax F5 (Molecular Devices). The glucose concentration was determined from the glucose standard curve.

### Triglyceride measurement

Triglyceride levels were quantified as previously described [90]. Five adult male flies were homogenized in 200 μl of PBS with Tween 20. The supernatant (30 μl) was used to measure the protein content using BCA assay kits. The samples were heated at 70°C for 10 min, and 40 μl of the samples were mixed with 40 μl of triglyceride reagent (T2449-10ML, Sigma). The mixture was incubated at 37°C for 45 min. After centrifugation, 30 μl of the supernatant was used to determine the TAG concentration by adding a free glycerol reagent (F6428, Sigma).

### Survival analysis

Two hundred male *Canton-S* flies (10 vials of 20 flies each) were collected for both ND-fed and HFD-fed groups. Flies at 0–4 days of age were placed on either ND or HFD and fed for 7 days. The number of deceased flies was counted daily over the 7 days for both diets. The survival rate was calculated using the following formula, and the average survival rate was determined.


Survival Rate=100×(20 - (number of flies that died))/20

### Glucose tolerance test

Adult female *Canton-S* flies were first subjected to 24 h of starvation on 1% agar, followed by hemolymph collection. The flies were then transferred to vials containing 10% glucose agar for 1 h, with subsequent re-starvation for either 0.5 or 2 h. Hemolymph samples were collected after each treatment. Glucose concentrations were measured at four time points: after starvation, 1 h of glucose exposure, and 0.5 and 2 h of re-starvation.

### Food intake

Adult female *Canton-S* flies were starved for 24 hours on 1% agar, then transferred to vials containing 10% glucose and 10% Blue Dye (TCI) in agar for 1 hour. After feeding, body samples were collected and homogenized in 500 µl of PBST. The homogenate was centrifuged at 13,000 rpm for 25 minutes, and the supernatant was transferred to a new tube and centrifuged again under the same conditions. The absorbance of the final supernatant was measured at 630 nm, and food intake was quantified based on a standard curve.

### Oil Red O staining

Fly guts were dissected in phosphate-buffered saline (PBS) on ice. Samples were fixed at room temperature in 4% paraformaldehyde (PFA) in PBS containing 0.1% Triton X-100 (PBST) for 30 min. After fixation, tissues were washed twice with PBS for 5 min each. A brief wash with 60% isopropanol in PBS was then performed for 1 min. The Oil Red O (ORO) (Nacalai Tesque) working solution was freshly prepared by diluting a previously prepared 0.1% ORO stock solution in isopropanol with distilled water at a 6:4 ratio. Guts were then incubated in 1 ml of the working solution at room temperature for 30 min in the dark. Following staining, samples were washed again with 60% isopropanol for 1 min, followed by two to three additional washes with PBS (5 min each) to remove excess dye. Finally, tissues were mounted in 75% glycerol for imaging.

### Western blotting

Frozen fly samples were homogenized in a RIPA buffer. After centrifugation at 15,000 rpm for 5 min at 4°C, the supernatants were collected and mixed with a 2 × sample buffer, then boiled at 98°C for 5 min. Equal amounts of samples were loaded onto 10–15% SDS-PAGE gels and transferred to PVDF membranes (Merck Millipore). The membranes were washed with Tris-buffered saline containing 0.1% Tween-20 (TBST), blocked with 0.3% skim milk in TBST for 1 h at room temperature (RT), and incubated overnight at 4°C in TBST containing 0.3% skim milk with the following primary antibodies: GABARAP (E1J4E) (1:1000, Cell Signaling Technology, #13733), Ref(2)p (1:1000, Abcam, ab178440), and Actin (1:1000, DSHB, #JL20). After three washes with TBST, the membranes were further incubated for 1 h with secondary anti-rabbit POD (1:20000, Jackson ImmunoResearch) or anti-mouse POD (1:20000, Jackson ImmunoResearch). Following three washes with TBST, the membrane was treated with ECL solution and chemiluminescence was detected using the Ez-Capture MG system (ATTO). Band intensities were quantified using ImageJ software (NIH).

### Immunohistochemistry

For immunohistochemistry in fly brains, male flies were dissected in 0.1 M phosphate-buffered saline containing 0.3% Triton-X 100 (PBST), pH 7.2. The brains were fixed with 4% PFA containing 0.3% Triton on ice and then fixed at RT for 20 min, followed by three 20 min washes in PBST at RT. After a 2-h blocking step in blocking buffer (PBST with 5% normal goat serum) at RT, the brains were incubated for 48 h with primary antibodies in a blocking buffer at 4°C under gentle agitation. After incubation with secondary antibodies for two days at 4°C, the brains were washed three times for 20 min in PBST and mounted in VECTASHIELD Mounting Medium for 24 h at 4°C. The following antibody dilutions were used for Confocal microscopy: Ref(2)p (1:1000, Abcam, ab178440), Alexa Fluor 647 (1:500, Invitrogen Molecular Probes, A21245), and nuclei were stained with DAPI (1:500) during incubation with the secondary antibody. For the imaging of LysoTracker Red in brains, male flies were dissected in PBS, incubated in 1 µM LysoTracker Red DND 99 (Thermo Fisher Scientific, L7528) for 20 min, washed with PBS, fixed in 4% PFA for 20 min, and mounted in PBS. Confocal imaging was performed immediately after mounting

### Autophagic flux assay

Five-day-old male flies expressing the tandem fluorescent-tagged autophagy reporter GFP-mCherry-Atg8a under the control of the *Elav*-*GS* driver were fed with ND or HFD supplemented with RU486 for 5 days. After treatment, flies were dissected in PBS. Brains were fixed with 4% PFA containing 0.3% Triton on ice and then fixed at RT for 20 min, followed by three 20 min washes in PBS at RT. After washing, brains were mounted in VECTASHIELD Mounting Medium for 12 h at 4°C.

### Confocal microscopy

Z-stack confocal images were captured using an LSM 780 confocal microscope (Zeiss) with the following objectives: Plan-Apochromat 63x/1.40 and 40x/1.30. Images shown in the same figure were acquired using the same gain from samples that were simultaneously fixed and stained. All images were captured around the cell bodies of the MBNs. Images were generated by collecting a stack of 25–30 images with focal planes 1.0 μm apart.

Confocal images were analyzed using Measure or Analyze Particles in the Fiji distribution of the ImageJ software, supplemented by calculations in Excel (Microsoft) and RStudio. The ImageJ threshold was used to analyze the number and area of positive signals. For Ref(2)p and LysoTracker Red counting, the middle layer of KCs was selected in the z-stack image, and the range of Ref(2)p-positive signals and LysoTracker puncta were selected using the ImageJ threshold. The analyzed particles were counted automatically. For the autophagy flux, three discontinuous layers, which contain similar KC layers between each individual and condition, were selected from approximately 30 layers of the z-stack images. The positive puncta of autophagosome and autolysosome within each layer were measured separately and averaged by using Excel.

### Quantitative real-time PCR

Total RNA was extracted from adult fly samples with Sepasol‐RNA I Super G (Nacalai Tesque), and the reverse-transcription reactions were performed with ReverTra Ace (TOYOBO). The mRNA expression levels were quantified using real-time PCR with the THUNDERBIRD SYBR qPCR Mix (TOYOBO) on a LightCycler 96 System (Roche). Expression levels were normalized relative to the expression of *rp49*. The primers used are shown below.

*atg1*: 5′-tgtcaccggatctgagggat-3′; 5′ -tggcatgtcaactggcgac-3′
*atg5*: 5′-tgcctcaccattcacttctcc-3′; 5′-gcttaagcacatctgcctcctt-3′
*rubicon*: 5′-ggcccaaagctgcactagaa-3′; 5′-ggcgtaatccggtcgataag-3′
*LManII*: 5′-tcagcgaatttgggagagga-3′; 5′-gtgcggtagtattgcgattgg-3′
*cp1*: 5′-acgaaaggtctttgggactgg-3′; 5′-cagctgtgcgcattgtgatt-3′
*sap-r*: 5′-accgggaaaatggagagagc-3′; 5′-cgacattccttggagttgctga-3′
*CG18858*: 5′-tggcgtcaattgagtagcgcga-3′; 5′-agcctcattgtgcgttgcgttg-3′
*CG4847*: 5′-acaccttcaagcaggccgtgaa-3′; 5′-tcgcaggcaggttgaccaactt-3′
*CG12163*: 5′-aagcaccgcgaactggttacct-3′; 5′-tcgttaagcagtcgcgccttct-3′
*CG11459*: 5′-acacactccgttcttttagtc-3′; 5′-agccactttctccccaatc-3′
*npc2h*: 5′-gagatagtcaactttcagact-3′; 5′-gcgcaccaccttgatgtc-3′
*rp49*: 5′-atcggttacggatcgaacaa-3′; 5′-gacaatctccttgcgcttct-3′

### RNA-seq and analysis

Total RNA was extracted from the heads of 10-day-old male flies fed either ND or HFD. 3′ RNA-seq experiments were performed at the Kazusa DNA Research Institute (Chiba, Japan). Differential gene expression analyses were conducted using the “DESeq2” package of R software, and genes were considered differentially expressed if p < 0.05. The “clusterprofile” package and “ggplot2” package of R software were used to analyze and visualize the KEGG analysis.

### Statistics

GraphPad Prism software ver. 8.4.3 (GraphPad Software Inc.) was used for statistical analysis. The behavioral assay results were expressed as the mean and SEM. Other results were expressed as the mean and SD. We first used normal and lognormal tests to analyze whether the data followed a normal distribution. If the data followed a normal distribution, they were analyzed using either one- or two-way ANOVA followed by indicated post hoc tests for multiple analyses (Student’s *t*-test for two groups and ANOVA for three groups or more). If the data did not follow a normal distribution, they were analyzed using the Mann–Whitney (for two groups) or Kruskal–Wallis test (for three groups or more). The confidence level of all tests was set at p < 0.05. The detailed explanation of the tests and comparisons made for each experiment are presented in each figure legend.

## Supporting information

S1 Fig HFD decreases intermediate-term memory. (A) The survival rate of 10d *Canton-S* fed with ND or HFD was observed for 7 days. There was no significant decline in the survival rate in flies fed with HFD compared with ND (n = 10 vials, containing 20 flies at each time point). (B) HFD feeding led to lipid accumulation in the gut, as revealed by Oil Red O staining. Scale bar: 70 µm. Error bars indicate SD (n = 3. Student’s *t*-test, * p < 0.05). (C) Food intake was not significantly different between ND- and HFD-fed flies during the 1 hour. (D) Electric shock sensitivity in flies fed with ND and HFD. Different diet-fed flies have similar shock avoidance. Error bars indicate SEM (n = 7, 6. Student’s *t*-test, n.s., not significant). (E) 3-Octanol (Oct) and Benzaldehyde (Benz) sensitivity in flies fed with ND and HFD. Different diets-fed flies have similar odor avoidance to Oct. However, HFD decreases flies’ sensitivity to Benz. Error bars indicate SEM (n = 18, 12, 15, 10. Two-way ANOVA, n.s., not significant, **** p < 0.0001). (F) 4-methylcyclohexanol (MCH) sensitivity in flies fed with ND and HFD. Different diets-fed flies have similar odor avoidance to MCH (n = 6. Student’s *t*-test, n.s., not significant). (G) Memory performance using MCH and Oct as olfactory cues. HFD feeding did not affect STM but significantly impaired ITM in flies (n = 12, 10, 13, 14. Two-way ANOVA, n.s., not significant, * p < 0.05).
(S1_Fig.TIF)

S2 Fig Transient suppression of *atg5* in neurons does not impair ITM. (A) Knockdown efficiency of Atg5 RNAi in the whole body. Error bars indicate SD (n = 3. Student’s *t*-test, **** p < 0.0001). (B) Knockdown efficiency of Atg5 RNAi in the neuron system. Error bars indicate SD (n = 3. One-way ANOVA, ** p < 0.01). (C) Oct sensitivity in Atg5 knockdown flies. Three groups of flies had similar odor avoidance to Oct. Error bars indicate SEM (n = 6. Kruskal-Wallis test, n.s., not significant). (D) Benz sensitivity in Atg5 knockdown flies. Three groups of flies had similar odor avoidance to Benz. Error bars indicate SEM (n = 4, 6, 6. One-way ANOVA, n.s., not significant). (E) Electric shock sensitivity in Atg5 knockdown flies. *Elav-Gal4/UAS-Atg5* RNAi flies had slightly weaker shock avoidance. Error bars indicate SEM (n = 6, 12, 6. One-way ANOVA, * p < 0.05). (F) Knockdown efficiency of Atg5 RNAi. Error bars indicate SD (n = 3. Two-way ANOVA, *** p < 0.001, **** p < 0.0001). (G) ITM in flies with transient knockdown of Atg5 in neurons (*Elav-GS/UAS-Atg5 RNAi* RU+) is not changed compared with other genotypes. Error bars indicate SEM (n = 10, 11, 10, 10. Two-way ANOVA, n.s., not significant).
(S2_Fig.TIF)

S3 Fig Knockdown of *atg1* in DPM neurons causes memory loss. (A) Knockdown efficiency of Atg1 RNAi. Error bars indicate SD (n = 3. Two-way ANOVA, * p < 0.05). (B) Oct sensitivity of *Elav-GS/+* and *Elav-GS/UAS-Atg1 RNAi* flies. These flies have similar odor avoidance to Oct. Error bars indicate SEM (n = 6. Two-way ANOVA, n.s., not significant). (C) Benz sensitivity of *Elav-GS/+* and *Elav-GS/UAS-Atg1 RNAi* flies. These flies have similar sensitivity to Benz. Error bars indicate SEM (n = 6, 6, 6, 8. Two-way ANOVA, n.s., not significant). (D)Electric shock sensitivity of *Elav-GS/+* and *Elav-GS/UAS-Atg1 RNAi* flies. These flies have similar shock avoidance. Error bars indicate SEM (n = 6, 6, 6, 8. Two-way ANOVA, n.s., not significant). (E) ITM was significantly impaired in *VT64246-Gal4/UAS-Atg1 RNAi* flies compared to control groups. Error bars indicate SEM (n = 10, 10. Student’s *t*-test, *** p < 0.001). (F) Benz sensitivity in *VT64246-Gal4/+* and *VT64246-Gal4/UAS-Atg1 RNAi* flies. These flies have similar sensitivity to Benz. Error bars indicate SEM (n = 6, 6. Student’s *t*-test, n.s., not significant). (G) Oct sensitivity in *VT64246-Gal4/+* and *VT64246-Gal4/UAS-Atg1 RNAi* flies. Experimental flies have lower sensitivity to Oct. Error bars indicate SEM (n = 6, 6. Student’s *t*-test, * p < 0.05). (H)Electric shock sensitivity of *VT64246-Gal4/+* and *VT64246-Gal4/UAS-Atg1 RNAi* flies. These flies have similar shock avoidance. Error bars indicate SEM (n = 6, 6. Student’s *t*-test, n.s., not significant).
(S3_Fig.TIF)

S4 Fig HFD decreases *rubicon* expression level. (A) qPCR measures *rubicon*’s relative expression levels in ND and HFD head samples (n = 6, 7. Student’s *t*-test, ** p < 0.01). (B) Knockdown efficiency of Rubicon RNAi in flies of *Elav-GS/+* or *Elav-GS/UAS-Rubicon RNAi*. Error bars indicate SD (n = 3. Student’s *t*-test, *** p < 0.001, ** p < 0.01). (C) Expression level of *atg1* in the transient overexpression of *atg1* group (*Elav-GS/UAS-Atg1*, RU+) and control groups in heads. Error bars indicate SD (n = 3. Two-way ANOVA, ** p < 0.01).
(S4_Fig.TIF)

S5 Fig Elevated autophagic activity leads to decreased Ref(2)p protein level. (A) The experimental timeline of RU feeding and immunohistochemistry in flies. (B) Representative images of Ref(2)p immunostaining in the MB of flies fed with RU. The genotypes of the flies are *Elav-GS/ + *, *Elav-GS/UAS-Rubicon RNAi*, and *Elav-GS/UAS-Atg1*. Confocal sections through the Kenyon cells of MB were shown. Ref(2)p was shown in magenta, and DAPI marked nucleus. Scale bar: 10 µm. (C) Quantification of Ref(2)p staining-positive puncta. Error bars indicate SD (n = 17, 10, 12. Kruskal-Wallis test, ** p < 0.01, **** p < 0.0001).
(S5_Fig.TIF)

S6 Fig Normal odor or shock acuity in Rubicon RNAi or Atg1 overexpression flies. (A) Oct sensitivity of *Elav-GS/+* and *Elav-GS/UAS-Rubicon RNAi* flies. These flies have similar odor avoidance to Oct. Error bars indicate SEM (n = 6. Two-way ANOVA, n.s., not significant). (B) Benz sensitivity of *Elav-GS/+* and *Elav-GS/UAS-Rubicon RNAi* flies. These flies have similar sensitivity to Benz. Error bars indicate SEM (n = 6. Two-way ANOVA, n.s., not significant). (C) Electric shock sensitivity of *Elav-GS/+* and *Elav-GS/UAS-Rubicon RNAi* flies. These flies have similar shock avoidance. Error bars indicate SEM (n = 6. Two-way ANOVA, n.s., not significant). (D) Oct sensitivity of *Elav-GS/+* and *Elav-GS/UAS-Atg1* flies. These flies have similar odor avoidance to Oct. Error bars indicate SEM (n = 6. Two-way ANOVA, n.s., not significant). (E) Benz sensitivity of *Elav-GS/+* and *Elav-GS/UAS-Atg1* flies. These flies have similar sensitivity to Benz. Error bars indicate SEM (n = 6. Two-way ANOVA, n.s., not significant). (F) Electric shock sensitivity of *Elav-GS/+* and *Elav-GS/UAS-Atg1* flies. These flies have similar shock avoidance. Error bars indicate SEM (n = 6. Two-way ANOVA, n.s., not significant).
(S6_Fig.TIF)

S7 Fig Expression levels of lysosome-related genes in HFD-fed flies and control group. (A-E) qPCR measures each lysosome-related gene’s relative expression levels in ND and HFD head samples. The normalized expression level is shown (n = 6. Mann-Whitney test for CG12163; other genes use Student’s *t*-test, n.s., not significant).
(S7_Fig.TIF)

S8 Fig Knockdown efficiency of lysosome-related genes in RNAi lines. (A) Knockdown efficiency of LManII RNAi. Error bars indicate SD (n = 3. Two-way ANOVA, ** p < 0.01, *** p < 0.001). (B) Knockdown efficiency of CG4847 RNAi. Error bars indicate SD (n = 3. Two-way ANOVA, * p < 0.05, ** p < 0.01). (C) Knockdown efficiency of Sap-r RNAi in the neuron system. Error bars indicate SD (n = 3, Two-way ANOVA, n.s., not significant). (D) Knockdown efficiency of Sap-r RNAi in whole body. Error bars indicate SD (n = 4. Student’s *t*-test, **** p < 0.0001). (E) Oct and Benz sensitivity in flies fed with ND and CQ. Different diet-fed flies have similar odor avoidance to odors. Error bars indicate SEM (n = 6. Mann-Whitney test, n.s., not significant). (F) Electric shock sensitivity in flies fed with ND and CQ. Different diet-fed flies have similar shock avoidance. Error bars indicate SEM (n = 6. Student’s *t*-test, n.s., not significant).
(S8_Fig.TIF)

S9 Fig Model for HFD-induced memory decline. HFD leads to ITM decline by inhibiting autophagic activity in flies’ brain. The model was created with BioRender.
(S9_Fig.TIF)

S1 Data Differential gene expression analysis between ND- and HFD-fed fly head samples. (S1_Data.XLSX)
