# Supplementary figures and images for: High-fat diet impairs intermediate-term memory by autophagic-lysosomal dysfunction in Drosophila
Source: PLoS Genet. 2025 Aug 18;21(8):e1011818. doi: 10.1371/journal.pgen.1011818 (PMC12370196; doi:10.1371/journal.pgen.1011818)

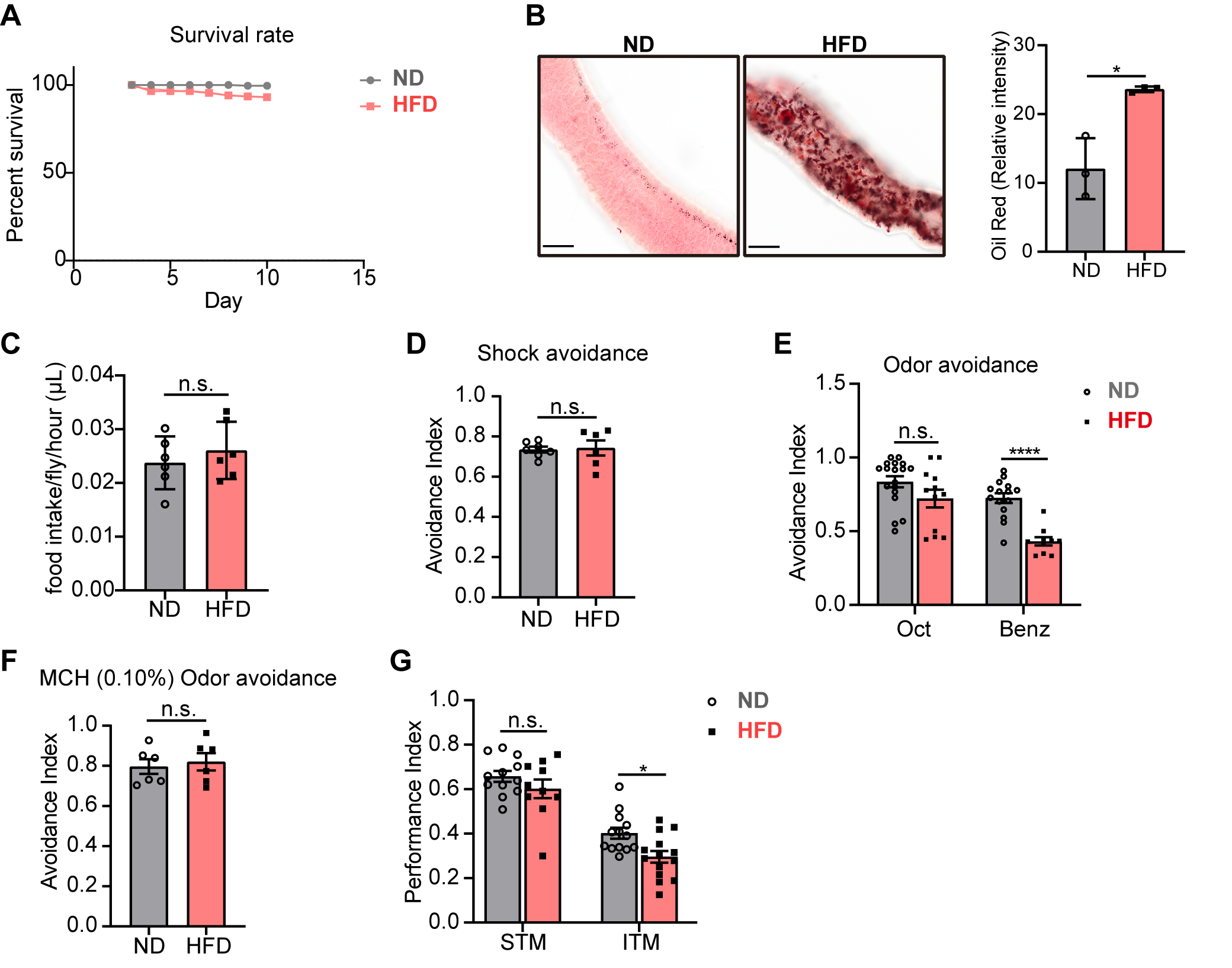

Supplement: S1 Fig — (A) The survival rate of 10d Canton-S fed with ND or HFD was observed for 7 days. There was no significant decline in the survival rate in flies fed with HFD compared with ND (n = 10 vials, containing 20 flies at each time point). (B) HFD feeding led to lipid accumulation in the gut, as revealed by Oil Red O staining. Scale bar: 70 µm. Error bars indicate SD (n = 3. Student’s t-test, * p < 0.05). (C) Food intake was not significantly different between ND- and HFD-fed flies during the 1 hour. (D) Electric shock sensitivity in flies fed with ND and HFD. Different diet-fed flies have similar shock avoidance. Error bars indicate SEM (n = 7, 6. Student’s t-test, n.s., not significant). (E) 3-Octanol (Oct) and Benzaldehyde (Benz) sensitivity in flies fed with ND and HFD. Different diets-fed flies have similar odor avoidance to Oct. However, HFD decreases flies’ sensitivity to Benz. Error bars indicate SEM (n = 18, 12, 15, 10. Two-way ANOVA, n.s., not significant, **** p < 0.0001). (F) 4-methylcyclohexanol (MCH) sensitivity in flies fed with ND and HFD. Different diets-fed flies have similar odor avoidance to MCH (n = 6. Student’s t-test, n.s., not significant). (G) Memory performance using MCH and Oct as olfactory cues. HFD feeding did not affect STM but significantly impaired ITM in flies (n = 12, 10, 13, 14. Two-way ANOVA, n.s., not significant, * p < 0.05). (S1_Fig.TIF) [file pgen.1011818.s001.tif]

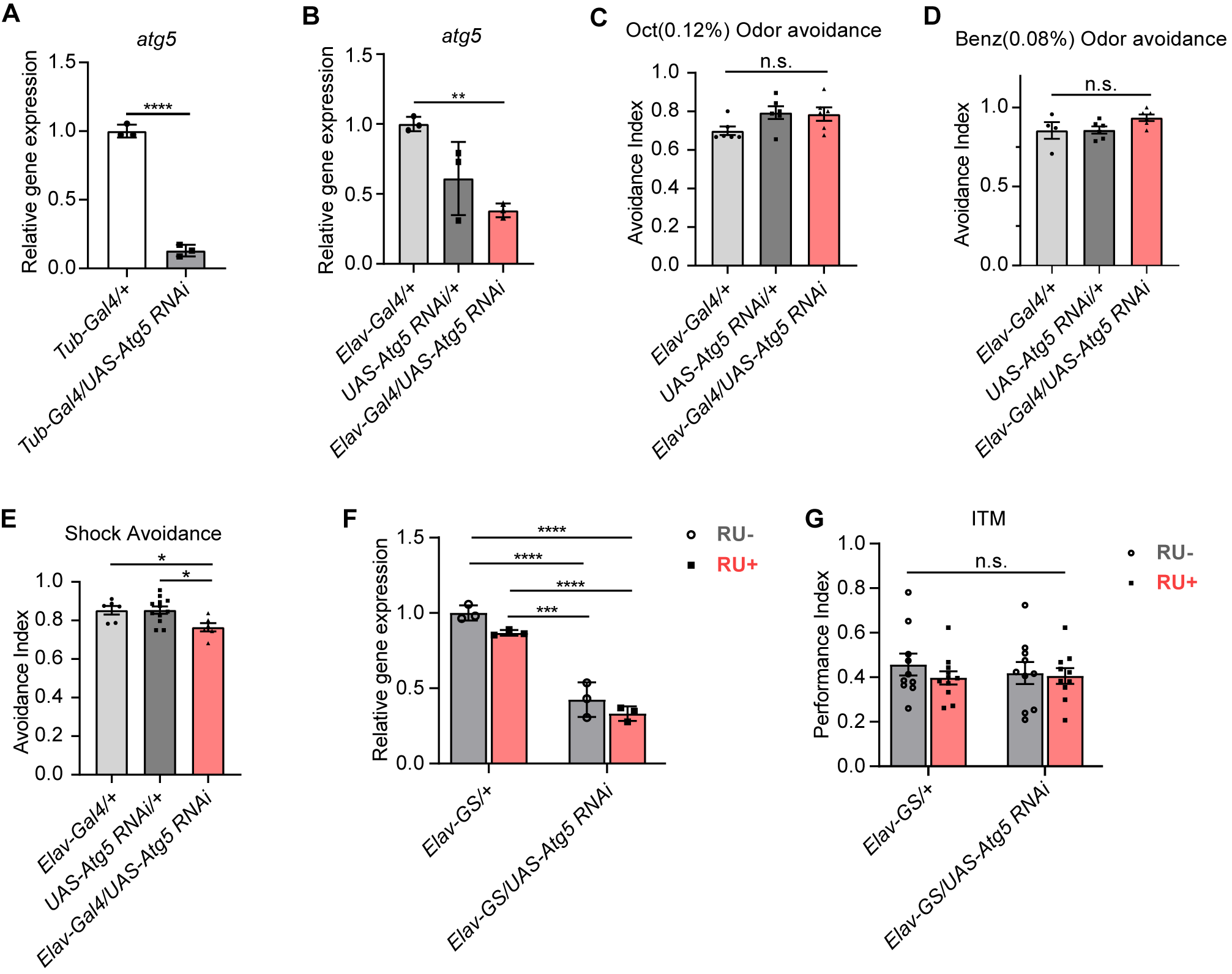

Supplement: S2 Fig — (A) Knockdown efficiency of Atg5 RNAi in the whole body. Error bars indicate SD (n = 3. Student’s t-test, **** p < 0.0001). (B) Knockdown efficiency of Atg5 RNAi in the neuron system. Error bars indicate SD (n = 3. One-way ANOVA, ** p < 0.01). (C) Oct sensitivity in Atg5 knockdown flies. Three groups of flies had similar odor avoidance to Oct. Error bars indicate SEM (n = 6. Kruskal-Wallis test, n.s., not significant). (D) Benz sensitivity in Atg5 knockdown flies. Three groups of flies had similar odor avoidance to Benz. Error bars indicate SEM (n = 4, 6, 6. One-way ANOVA, n.s., not significant). (E) Electric shock sensitivity in Atg5 knockdown flies. Elav-Gal4/UAS-Atg5 RNAi flies had slightly weaker shock avoidance. Error bars indicate SEM (n = 6, 12, 6. One-way ANOVA, * p < 0.05). (F) Knockdown efficiency of Atg5 RNAi. Error bars indicate SD (n = 3. Two-way ANOVA, *** p < 0.001, **** p < 0.0001). (G) ITM in flies with transient knockdown of Atg5 in neurons (Elav-GS/UAS-Atg5 RNAi RU+) is not changed compared with other genotypes. Error bars indicate SEM (n = 10, 11, 10, 10. Two-way ANOVA, n.s., not significant). (S2_Fig.TIF) [file pgen.1011818.s002.tif]

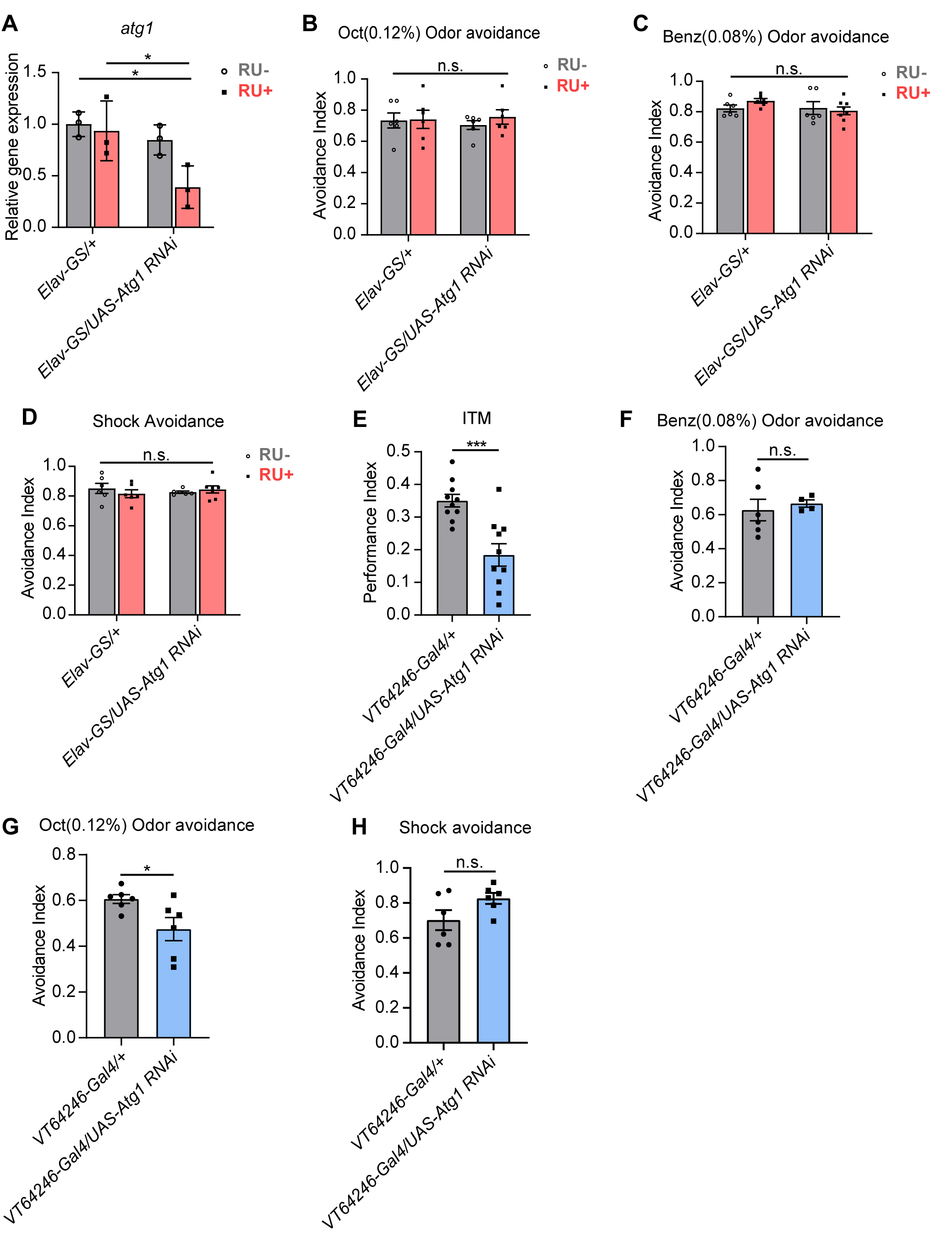

Supplement: S3 Fig — (A) Knockdown efficiency of Atg1 RNAi. Error bars indicate SD (n = 3. Two-way ANOVA, * p < 0.05). (B) Oct sensitivity of Elav-GS/+ and Elav-GS/UAS-Atg1 RNAi flies. These flies have similar odor avoidance to Oct. Error bars indicate SEM (n = 6. Two-way ANOVA, n.s., not significant). (C) Benz sensitivity of Elav-GS/+ and Elav-GS/UAS-Atg1 RNAi flies. These flies have similar sensitivity to Benz. Error bars indicate SEM (n = 6, 6, 6, 8. Two-way ANOVA, n.s., not significant). (D)Electric shock sensitivity of Elav-GS/+ and Elav-GS/UAS-Atg1 RNAi flies. These flies have similar shock avoidance. Error bars indicate SEM (n = 6, 6, 6, 8. Two-way ANOVA, n.s., not significant). (E) ITM was significantly impaired in VT64246-Gal4/UAS-Atg1 RNAi flies compared to control groups. Error bars indicate SEM (n = 10, 10. Student’s t-test, *** p < 0.001). (F) Benz sensitivity in VT64246-Gal4/+ and VT64246-Gal4/UAS-Atg1 RNAi flies. These flies have similar sensitivity to Benz. Error bars indicate SEM (n = 6, 6. Student’s t-test, n.s., not significant). (G) Oct sensitivity in VT64246-Gal4/+ and VT64246-Gal4/UAS-Atg1 RNAi flies. Experimental flies have lower sensitivity to Oct. Error bars indicate SEM (n = 6, 6. Student’s t-test, * p < 0.05). (H)Electric shock sensitivity of VT64246-Gal4/+ and VT64246-Gal4/UAS-Atg1 RNAi flies. These flies have similar shock avoidance. Error bars indicate SEM (n = 6, 6. Student’s t-test, n.s., not significant). (S3_Fig.TIF) [file pgen.1011818.s003.tif]

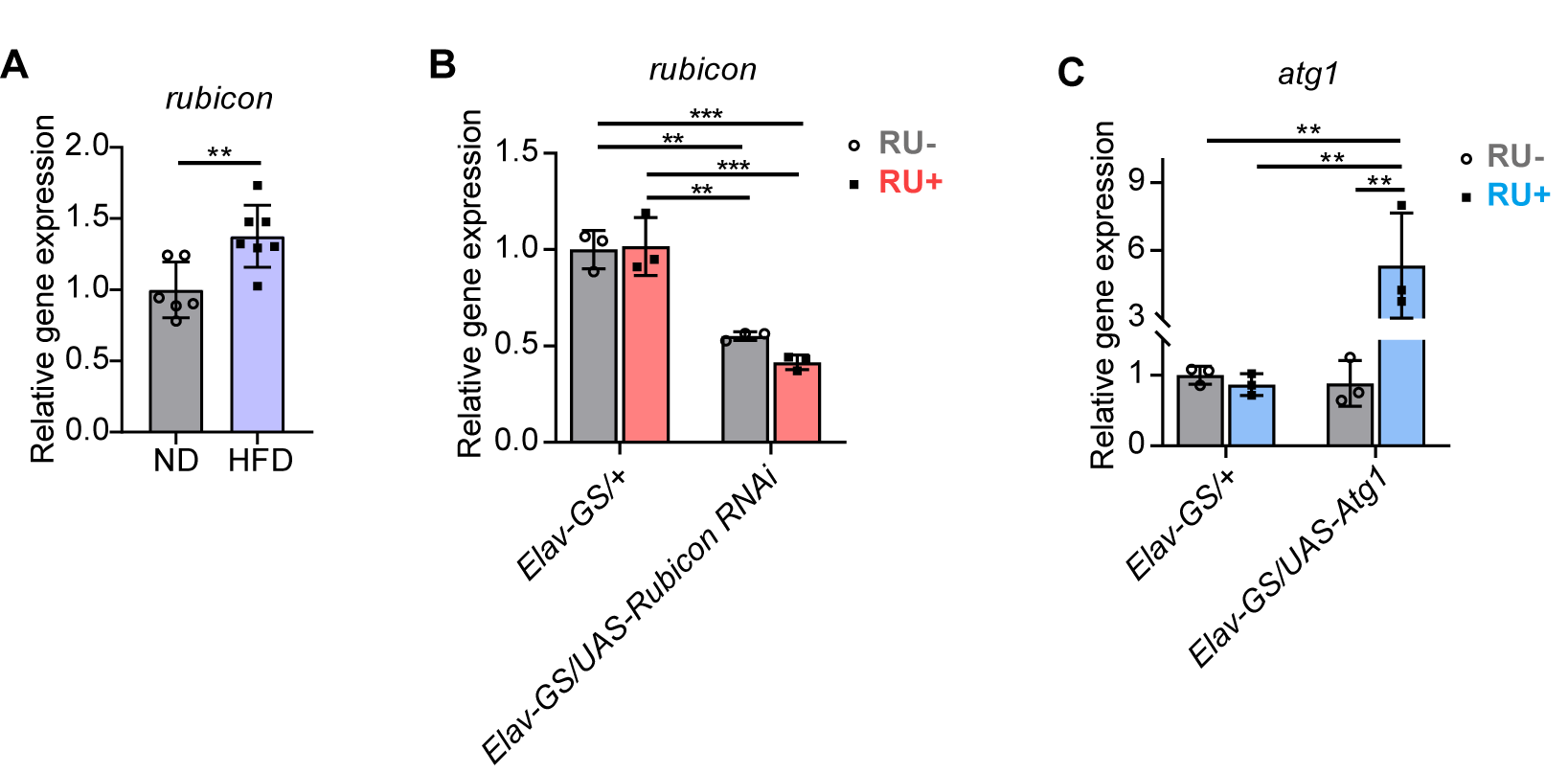

Supplement: S4 Fig — (A) qPCR measures rubicon’s relative expression levels in ND and HFD head samples (n = 6, 7. Student’s t-test, ** p < 0.01). (B) Knockdown efficiency of Rubicon RNAi in flies of Elav-GS/+ or Elav-GS/UAS-Rubicon RNAi. Error bars indicate SD (n = 3. Student’s t-test, *** p < 0.001, ** p < 0.01). (C) Expression level of atg1 in the transient overexpression of atg1 group (Elav-GS/UAS-Atg1, RU+) and control groups in heads. Error bars indicate SD (n = 3. Two-way ANOVA, ** p < 0.01). (S4_Fig.TIF) [file pgen.1011818.s004.tif]

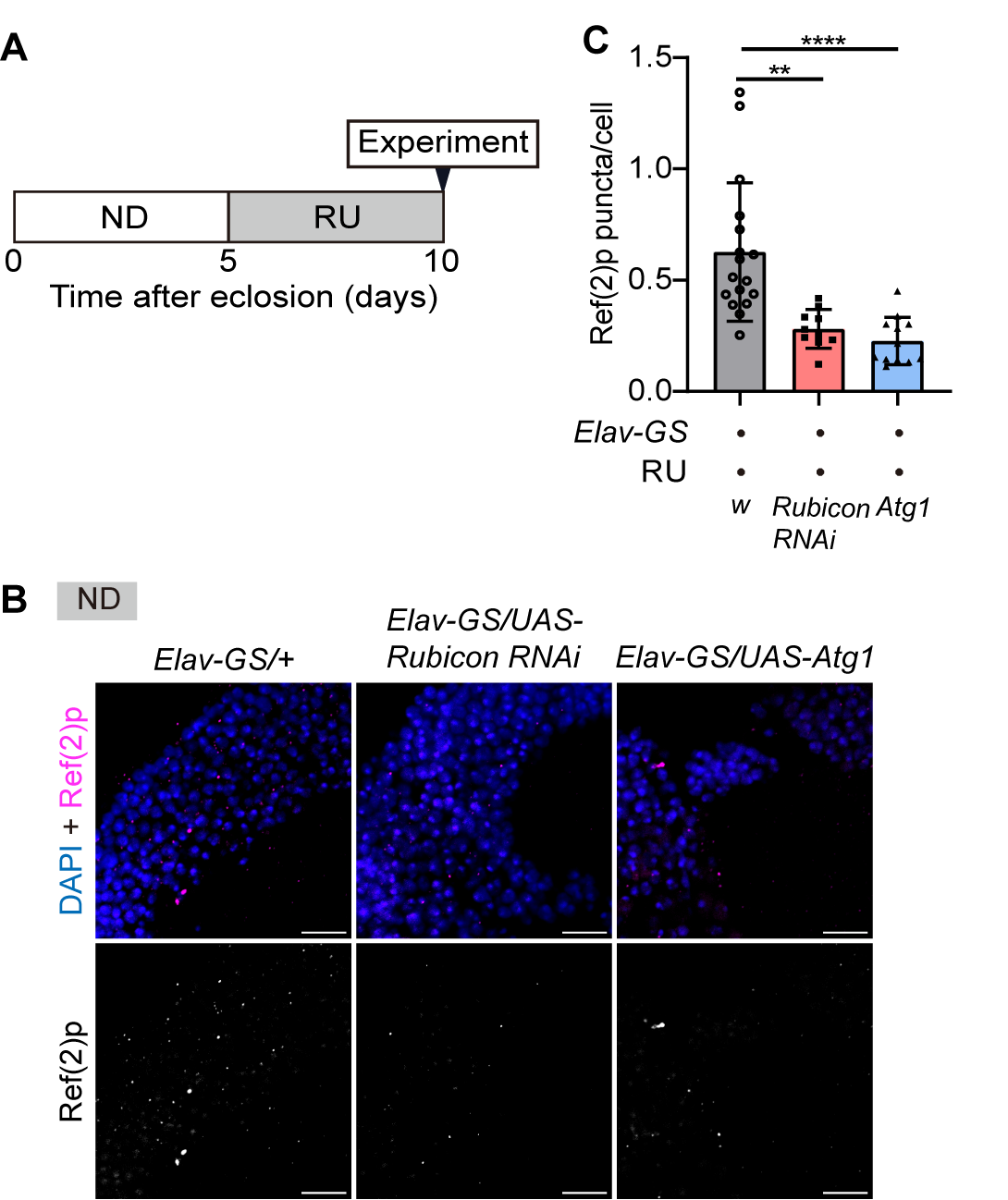

Supplement: S5 Fig — (A) The experimental timeline of RU feeding and immunohistochemistry in flies. (B) Representative images of Ref(2)p immunostaining in the MB of flies fed with RU. The genotypes of the flies are Elav-GS/ + , Elav-GS/UAS-Rubicon RNAi, and Elav-GS/UAS-Atg1. Confocal sections through the Kenyon cells of MB were shown. Ref(2)p was shown in magenta, and DAPI marked nucleus. Scale bar: 10 µm. (C) Quantification of Ref(2)p staining-positive puncta. Error bars indicate SD (n = 17, 10, 12. Kruskal-Wallis test, ** p < 0.01, **** p < 0.0001). (S5_Fig.TIF) [file pgen.1011818.s005.tif]

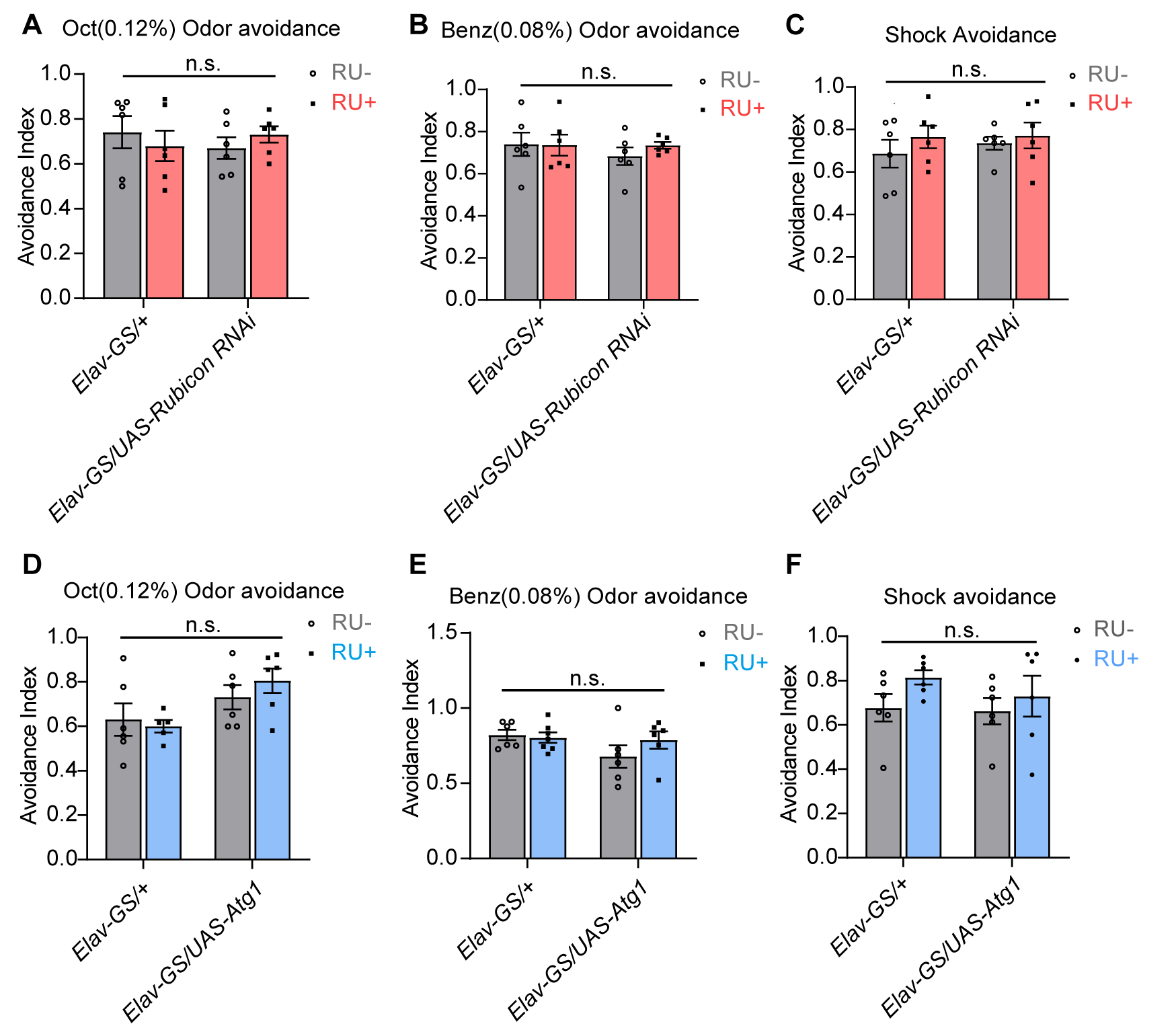

Supplement: S6 Fig — (A) Oct sensitivity of Elav-GS/+ and Elav-GS/UAS-Rubicon RNAi flies. These flies have similar odor avoidance to Oct. Error bars indicate SEM (n = 6. Two-way ANOVA, n.s., not significant). (B) Benz sensitivity of Elav-GS/+ and Elav-GS/UAS-Rubicon RNAi flies. These flies have similar sensitivity to Benz. Error bars indicate SEM (n = 6. Two-way ANOVA, n.s., not significant). (C) Electric shock sensitivity of Elav-GS/+ and Elav-GS/UAS-Rubicon RNAi flies. These flies have similar shock avoidance. Error bars indicate SEM (n = 6. Two-way ANOVA, n.s., not significant). (D) Oct sensitivity of Elav-GS/+ and Elav-GS/UAS-Atg1 flies. These flies have similar odor avoidance to Oct. Error bars indicate SEM (n = 6. Two-way ANOVA, n.s., not significant). (E) Benz sensitivity of Elav-GS/+ and Elav-GS/UAS-Atg1 flies. These flies have similar sensitivity to Benz. Error bars indicate SEM (n = 6. Two-way ANOVA, n.s., not significant). (F) Electric shock sensitivity of Elav-GS/+ and Elav-GS/UAS-Atg1 flies. These flies have similar shock avoidance. Error bars indicate SEM (n = 6. Two-way ANOVA, n.s., not significant). (S6_Fig.TIF) [file pgen.1011818.s006.tif]

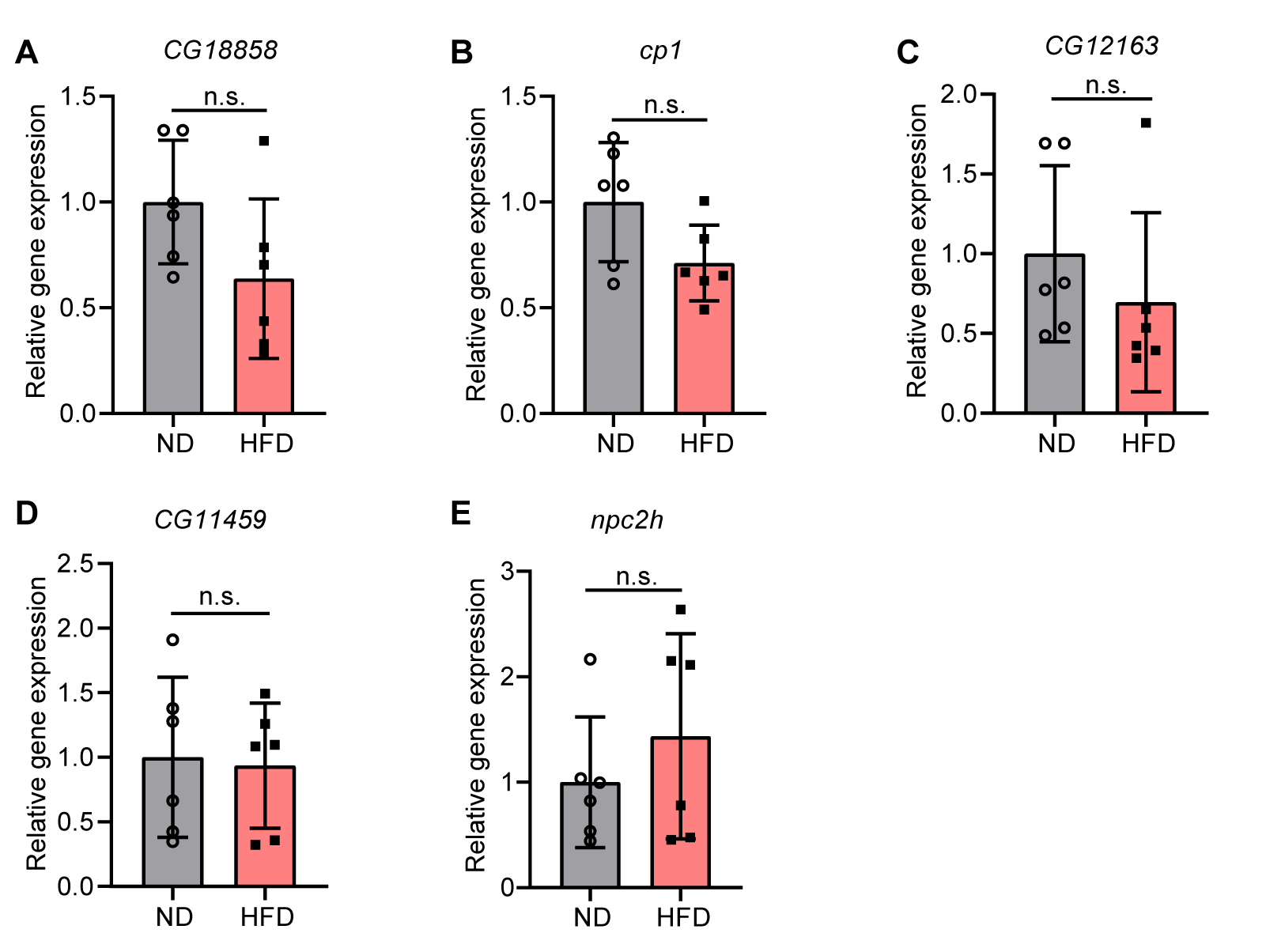

Supplement: S7 Fig — (A-E) qPCR measures each lysosome-related gene’s relative expression levels in ND and HFD head samples. The normalized expression level is shown (n = 6. Mann-Whitney test for CG12163; other genes use Student’s t-test, n.s., not significant). (S7_Fig.TIF) [file pgen.1011818.s007.tif]

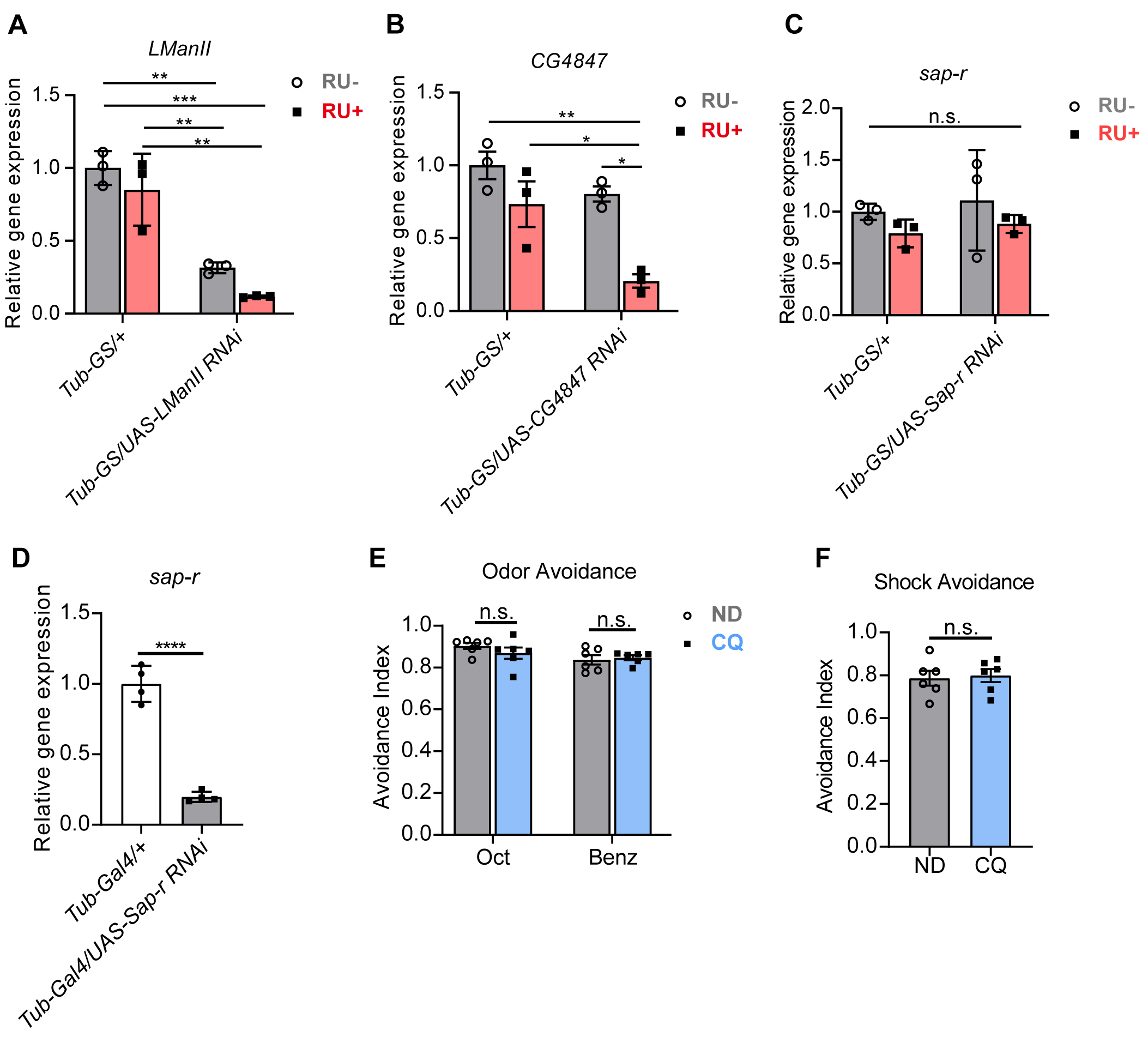

Supplement: S8 Fig — (A) Knockdown efficiency of LManII RNAi. Error bars indicate SD (n = 3. Two-way ANOVA, ** p < 0.01, *** p < 0.001). (B) Knockdown efficiency of CG4847 RNAi. Error bars indicate SD (n = 3. Two-way ANOVA, * p < 0.05, ** p < 0.01). (C) Knockdown efficiency of Sap-r RNAi in the neuron system. Error bars indicate SD (n = 3, Two-way ANOVA, n.s., not significant). (D) Knockdown efficiency of Sap-r RNAi in whole body. Error bars indicate SD (n = 4. Student’s t-test, **** p < 0.0001). (E) Oct and Benz sensitivity in flies fed with ND and CQ. Different diet-fed flies have similar odor avoidance to odors. Error bars indicate SEM (n = 6. Mann-Whitney test, n.s., not significant). (F) Electric shock sensitivity in flies fed with ND and CQ. Different diet-fed flies have similar shock avoidance. Error bars indicate SEM (n = 6. Student’s t-test, n.s., not significant). (S8_Fig.TIF) [file pgen.1011818.s008.tif]

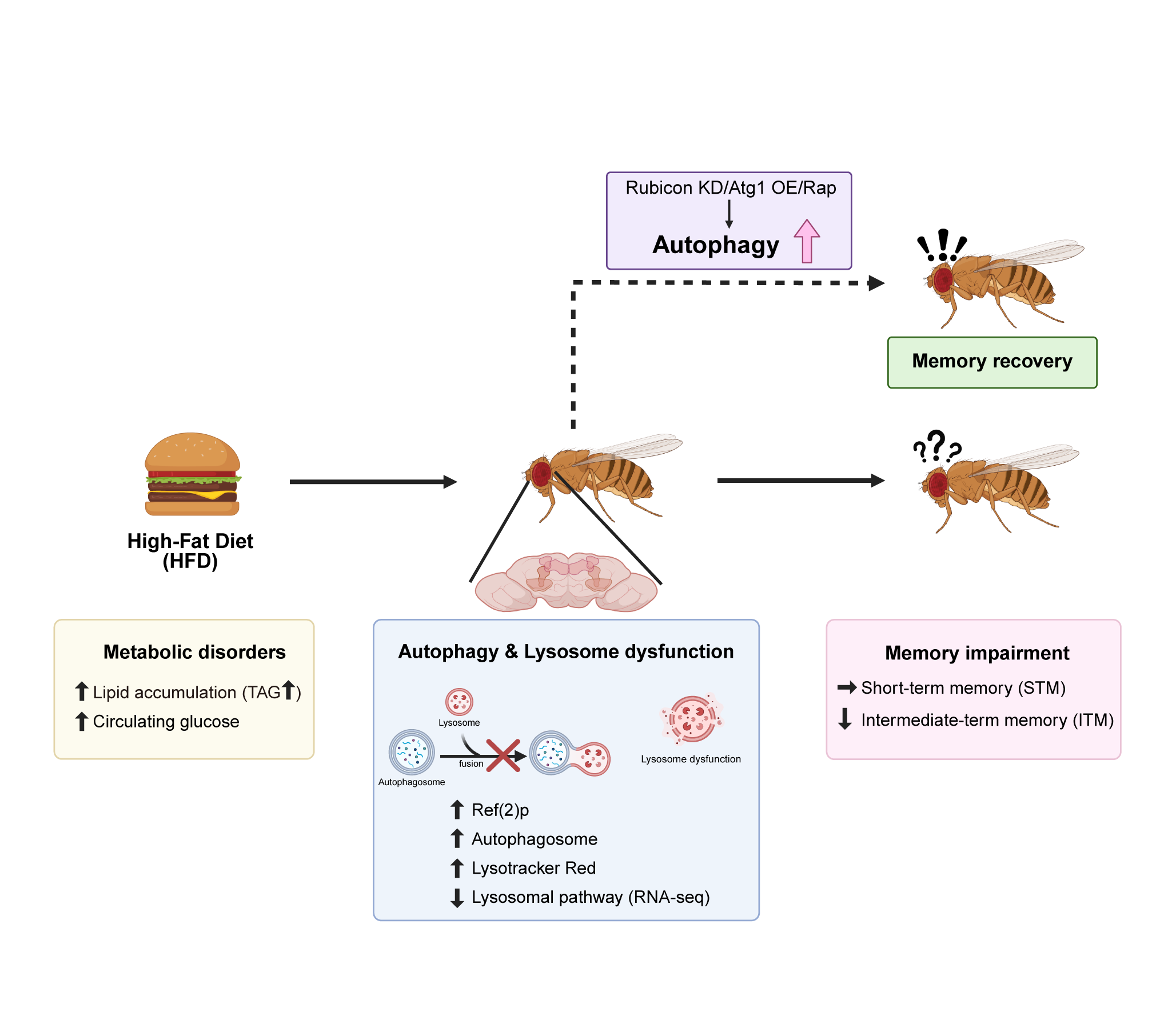

Supplement: S9 Fig — HFD leads to ITM decline by inhibiting autophagic activity in flies’ brain. The model was created with BioRender. (S9_Fig.TIF) [file pgen.1011818.s009.tif]
